# Interactions of Zinc Oxide Nanostructures with Mammalian Cells: Cytotoxicity and Photocatalytic Toxicity

**DOI:** 10.3390/ijms21176305

**Published:** 2020-08-31

**Authors:** Chengzhu Liao, Yuming Jin, Yuchao Li, Sie Chin Tjong

**Affiliations:** 1 Department of Materials Science and Engineering, Southern University of Science and Technology, Shenzhen 518055, China; liaocz@sustech.edu.cn (C.L.); jinym@mail.sustech.edu.cn (Y.J.); 2 Department of Materials Science and Engineering, Liaocheng University, Liaocheng 252000, China; 3 Department of Physics, City University of Hong Kong, Tat Chee Avenue, Kowloon, Hong Kong, China

**Keywords:** Zinc Oxide, metal doping, plasmonic nanoparticle, oxidative stress, zincate, photodynamic therapy, ultraviolet light, cancer cell, mouse model

## Abstract

This article presents a state-of-the-art review and analysis of literature studies on the morphological structure, fabrication, cytotoxicity, and photocatalytic toxicity of zinc oxide nanostructures (nZnO) of mammalian cells. nZnO with different morphologies, e.g., quantum dots, nanoparticles, nanorods, and nanotetrapods are toxic to a wide variety of mammalian cell lines due to in vitro cell–material interactions. Several mechanisms responsible for in vitro cytotoxicity have been proposed. These include the penetration of nZnO into the cytoplasm, generating reactive oxygen species (ROS) that degrade mitochondrial function, induce endoplasmic reticulum stress, and damage deoxyribonucleic acid (DNA), lipid, and protein molecules. Otherwise, nZnO dissolve extracellularly into zinc ions and the subsequent diffusion of ions into the cytoplasm can create ROS. Furthermore, internalization of nZnO and localization in acidic lysosomes result in their dissolution into zinc ions, producing ROS too in cytoplasm. These ROS-mediated responses induce caspase-dependent apoptosis via the activation of B-cell lymphoma 2 (Bcl2), Bcl2-associated X protein (Bax), CCAAT/enhancer-binding protein homologous protein (chop), and phosphoprotein p53 gene expressions. In vivo studies on a mouse model reveal the adverse impacts of nZnO on internal organs through different administration routes. The administration of ZnO nanoparticles into mice via intraperitoneal instillation and intravenous injection facilitates their accumulation in target organs, such as the liver, spleen, and lung. ZnO is a semiconductor with a large bandgap showing photocatalytic behavior under ultraviolet (UV) light irradiation. As such, photogenerated electron–hole pairs react with adsorbed oxygen and water molecules to produce ROS. So, the ROS-mediated selective killing for human tumor cells is beneficial for cancer treatment in photodynamic therapy. The photoinduced effects of noble metal doped nZnO for creating ROS under UV and visible light for killing cancer cells are also addressed.

## 1. Introduction

**Figure 1:**
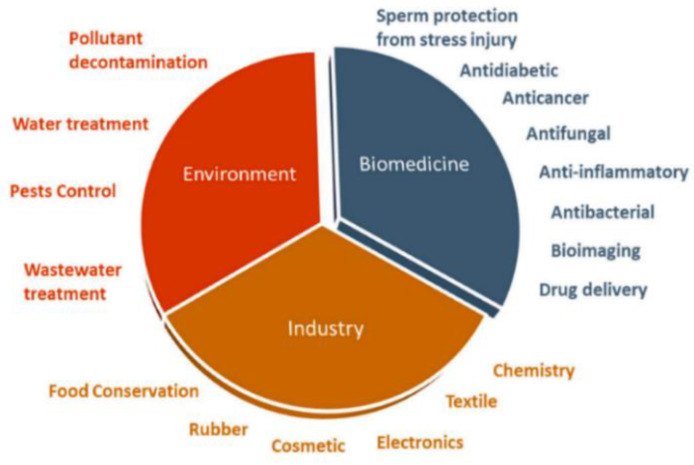
A wide variety of applications of ZnO nanoparticles in biomedicine, industry, and environment. Reproduced from [21] with permission of MDPI under the terms of the Creative Commons Attribution license.

Recent advances of nanotechnology in materials science have led to the development of novel materials of various types at the nanoscale level. Nanomaterials can be categorized into zero-dimensional (0-D), one-dimensional (1-D), two-dimensional (2-D), and three-dimensional (3-D) based on their dimensionality and shape. Typical examples are carbon quantum dots (0-D), carbon or titania nanotubes (1-D), graphene sheets (2-D), and zinc oxide nanoflowers (3-D). Those functional materials can be fabricated at nanometer-scale control and precision. Nanomaterials with unique biological, chemical, and physical properties are promising for applications in energy, agricultural, biomedical, environmental, industrial, and pharmaceutical sectors [1,2,3,4,5,6,7,8,9,10,11,12,13,14,15]. For applications in the biomedical field, nanomaterials are widely used in biosensing, bioimaging, antibacterial agents, drug delivery, and theranostics [16,17,18]. Semiconducting oxide nanomaterials based on titanium dioxide and zinc oxide are very effective for water treatment and purification due to their large surface area and photocatalytic effect [19,20]. In particular, zinc oxide (ZnO) finds attractive applications in biomedical field, environment and industry ranging from electronics, textiles, tires, cosmetics, food processing and preservation, etc. (Figure 1) [21]. Zinc oxide in the bulk form is considered as a “generally recognized as safe” (GRAS) substance by the United States Food and Drug Administration (FDA) [22]. Accordingly, ZnO has been incorporated into cosmetic and healthcare products, including toothpaste, sunscreens, and textile coatings. 

**Figure 2:**
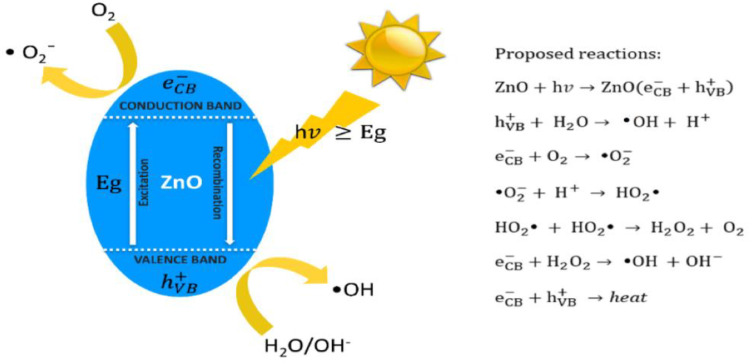
Schematic representation showing the creation of reactive oxygen species on ZnO by irradiating with photons (*hv*) having energies ≥ E_g_. Proposed photocatalytic reactions are listed in the right panel. Reproduced from [26] permission under the conditions of the Creative Commons Attribution (CC BY) license.

ZnO is an n-type semiconductor with a wide bandgap energy (Eg) of 3.37 eV, and contains intrinsic defects such as zinc interstitials and oxygen vacancies [23,24]. ZnO also possesses high dielectric permittivity, large excition binding energy, good chemical stability and excellent photocatalytic property [25]. These properties render ZnO an attractive material for fabricating semiconducting and optoelectronic devices. ZnO with a wide bandgap absorbs ultraviolet (UV) radiation, generating reactive oxygen species (ROS) such as hydroxyl (•OH), superoxide anion (•O_2_^−^), singlet oxygen (^1^O_2_), and hydrogen peroxide (H_2_O_2_) by reacting with adsorbed water/hydroxyl and oxygen molecules (Figure 2) [26]. By irradiating ZnO with UV light with an energy larger than Eg, electron in the valence band (VB) of ZnO promotes to the conduction band (CB), thus producing a positively charged hole in the VB, i.e., h_VB_^+^. The electron–hole pair then moves to the surface of ZnO, and reacts with adsorbed water and oxygen molecules to generate •O_2_^−^ and •OH accordingly. Generally, short-lived superoxide and hydroxyl radicals on photocatalysts can be measured directly by means of electron spin resonance (ESR) spectroscopy [27].

As mentioned, ZnO has been used widely in various applications including sunscreens, toothpastes, food additives, beverages, food packaging films, solar cells, coatings, photocatalysts, medical textiles, etc. So, safety issues relating the widespread use of ZnO nanoparticles (NPs) have raised public concern about their potential toxicity for humans and animals [28]. ZnO NPs can enter human body through several routes including skin penetration, inhalation, digestive system and parenteral injection. Safety assessment of ZnO NPs in sunscreens through the skin contact has been carried out by the researchers [29,30]. More recently, Roberts and coworkers reported that ZnO NPs in sunscreens accumulated on the skin surface and within the skin furrows of human volunteers. ZnO NPs did not penetrate into the viable epidermis and induced toxicity in the underlying viable epidermis [30]. Comparing with sunscreen consumers, the workers in the ZnO NPs manufacturing plants would inhale and contact dermally with a larger amount of these nanoparticles during the production process [31]. The respiratory tract is the main entry pathway for direct exposure of ZnO NPs. The nanoparticles then deposit on alveolar epithelial cells and induce pulmonary inflammatory response accordingly [32]. Apart from pulmonary damage, increased exposure to ZnO NPs would lead to hepatotoxicity and male reproductive toxicity through the induction of endoplasmic reticulum (ER) stress [33,34,35]. As recognized, ER is an organelle responsible for protein folding. Accumulation of misfolded proteins in the ER could lead to ER stress [36].

Nowadays, environmental pollution and ageing population cause a significant rise in the number of patients suffering from tumors. Apart from radiotherapy and chemotherapy, photodynamic therapy (PDT) has become a viable option for surgeons and radiologists for treating cancer patients. PDT treatment involves the generation of ROS from a photosensitizer injected into human body under a suitable excitation light source [37]. Semiconductor oxide nanoparticles such as ZnO NPs and TiO_2_ NPs capable of generating ROS under UV light for cancer treatment is particularly attractive for the PDT [38,39]. This article provides a state-of-the art review of the reported studies on the cytotoxicity and photocatalytic toxicity of ZnO nanostructures in mammalian cells. The nZnO-induced toxicity mediated by ROS generation under UV irradiation for human cancer cell therapy is also addressed.

## 2. Structure-Dependent Photocatalytic Activity

### 2.1. Lattice Structure

**Figure 3:**
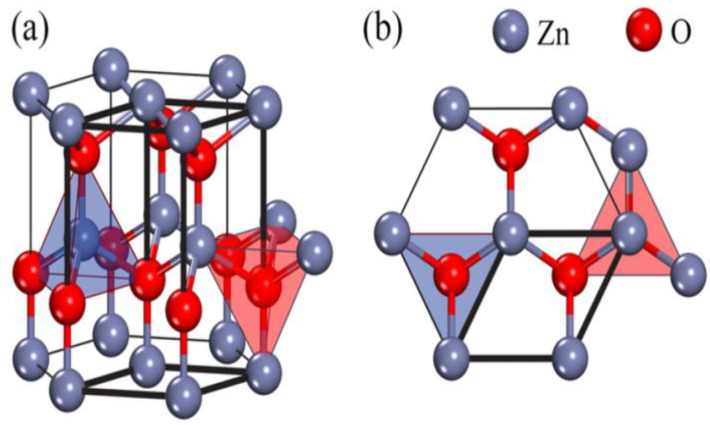
(**a**) Side view and (**b**) top view of ZnO wurtzite structure. Reproduced from [43] with permission of Elsevier.

**Figure 4:**
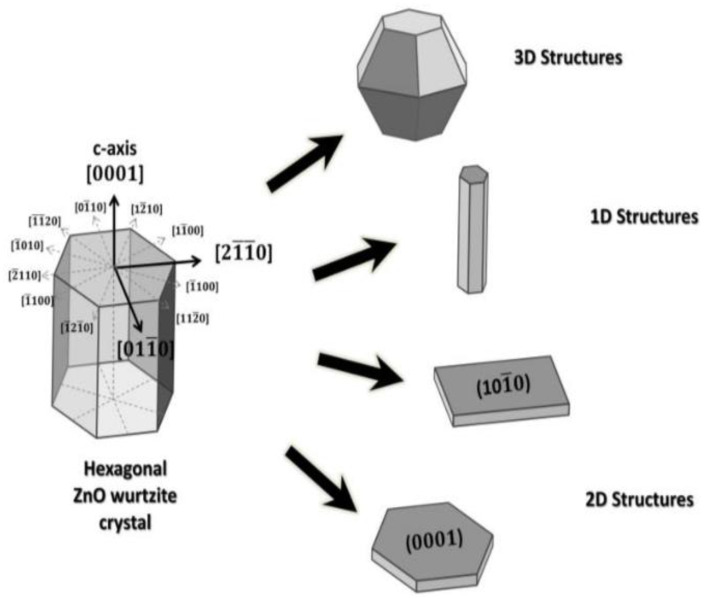
ZnO nanostructures with different dimensions. Reproduced from [46] under the Creative Commons Attribution license.

The crystalline structure of ZnO plays an important role in regulating photocatalytic activity. This in turn affects the generation of ROS on its surface. ZnO exists in three crystalline forms, namely hexagonal wurtzite, cubic rock salt (NaCl), and cubic zinc blende (metastable) [40,41]. The most stable structure is hexagonal wurtzite with the lattice parameters a = 0.3249 nm and c = 0.5205 nm [42]. In this structure, O^2−^ and Zn^2+^ create alternating planes of tetrahedral coordinated units, stacking along the c-axis (Figure 3a,b) [43]. This arrangement yields positively charged Zn-(0001) and negatively charged O-(000 $\bar{1}$) polar planes, resulting in spontaneous polarization and producing a dipole moment along the c-axis. Wurtzite ZnO undergoes a phase transformation to rocksalt structure at a high pressure of 9.1 GPa [44]. ZnO nanomaterials can be typically categorized into four groups based on their geometries and dimensions, i.e., 0D (quantum dot, nanoparticle), 1D (nanorod, nanotube, nanowire), 2D (nanosheet, nanoplate, nanodisk) and 3D (tetrapod, nanoflower) structures (Figure 4) [45,46,47,48]. In particular, 3D hierarchical nanostructure formation derives from the self-assembly of primary nanocrystals such as nanoparticles and nanorods during the synthesis process. In general, ZnO wurtzite phase exhibits three specific types of fast growth orientations, i.e., <0001>, <01 $\bar{1}$0>, and <2 $\bar{1}$ $\bar{1}$0>. In this respect, a wide variety of ZnO nanostructures can be prepared by monitoring the growth rates along those orientations [45,46,47,48]. The resulting nZnO show distinct biomedical activity, cytotoxicity, and application [49,50,51,52,53,54,55,56]. For instance, ZnO quantum dots can be employed as imaging nanoprobes for targeting cancer cells in vitro [51]. ZnO nanoflowers serve as effective drug delivery vehicles for biomedical applications [55].

### 2.2. Photocatalytic ROS Production

Photoinduced charge carriers (electrons and holes) in ZnO under UV irradiation recombine readily with the release of energy in the form of light or heat (Figure 2). The fast recombination of photoinduced charge carriers under UV light, and low visible light absorption limit the application of ZnO as a photocatalyst. Recombination of charge carriers can be suppressed by trapping photoinduced electrons or holes through the induction of surface vacancy defects and construction of Schottky junctions. So, the low optical absorption of ZnO in the visible light can be enhanced through the formation of oxygen vacancies, noble metal doping, non-metal doping, carbon nanomaterial modification, etc. Among these, noble metal doping is increasingly explored in biomedical sector to induce ROS on nZnO for cancer therapy. So, the ROS generation on nZnO due to oxygen vacancy induction and noble metal doping under visible light is briefly discussed herein.

**Figure 5:**
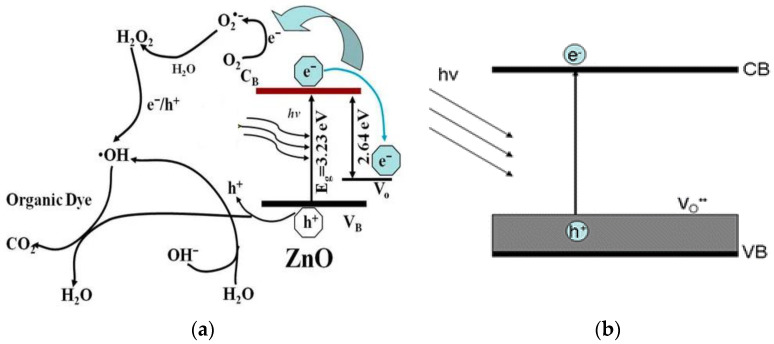
(**a**) Oxygen vacancies serve as the trap for photoinduced electrons under UV light, retarding charge carrier recombination and creating sufficient ROS for degrading organic dye. Reproduced from [58] under the Commons Attribution license. (**b**) Oxygen vacancies induce bandgap narrowing, facilitating the generation of electron–hole pair under visible light. Reproduced from [59] with permission of the American Chemical Society.

As mentioned, semiconducting ZnO contains intrinsic oxygen vacancy (V_o_) defects. Higher oxygen vacancy concentrations can be induced in ZnO by annealing in an inert environment or oxygen deficient atmosphere [57,58]. Those vacancies introduce midgap state above the VB of ZnO, providing the trapping site for photoinduced electrons under UV irradiation. Thus, the recombination of photoinduced electron–hole pair under UV light can be greatly retarded (Figure 5a). As a result, the induced ROS are very effective to degrade methylene blue (organic dye) [58]. In addition, the corresponding bandgap narrowing due to oxygen vacancies improves the optical absorption of ZnO under visible light, facilitating the excitation of electron–hole pairs and the production of ROS accordingly (Figure 5b) [59].

**Figure 6:**
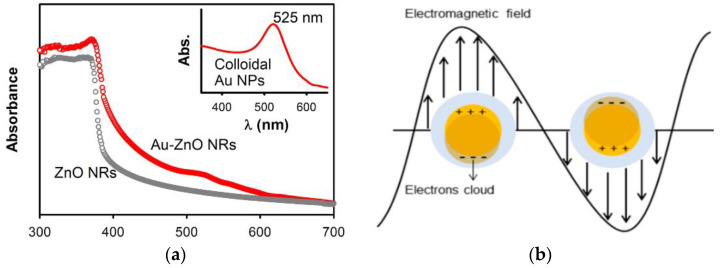
(**a**) UV/visible optical absorption spectra of pure ZnO nanorods (NRs) and AuNPs-doped ZnO NRs. Inset shows the UV/Vis spectrum of colloidal AuNPs (~20 nm) with a plasmon absorption peak at 525 nm. Reproduced from [61] with permission of Nature under the Creative Commons Attribution license. (**b**) Schematic illustration of oscillation of free electrons of noble metal NPs with electric field of incident light. Reproduced from [68] under the Creative Commons Attribution license.

**Figure 7:**
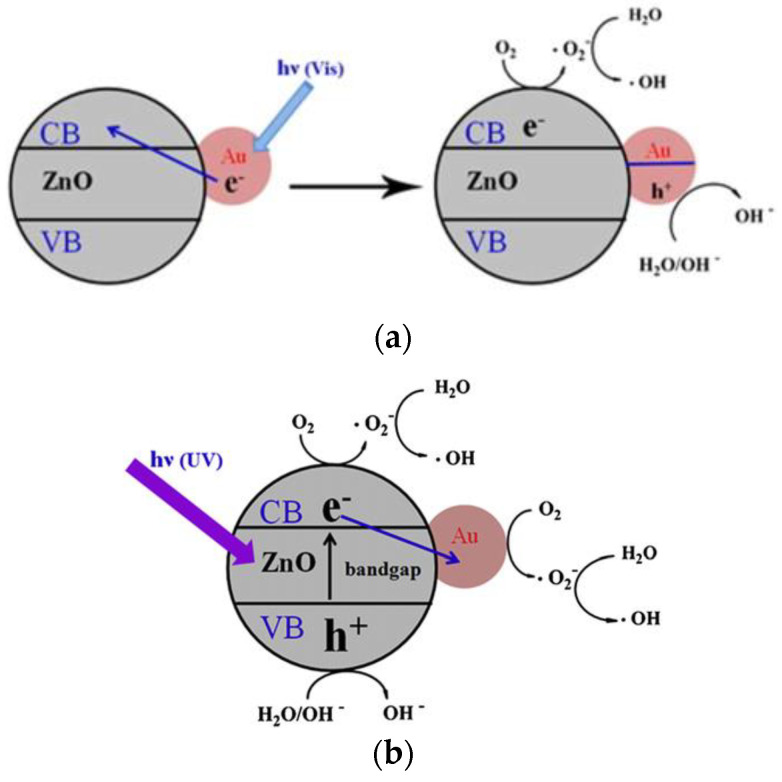
(**a**) Surface plasmon resonance (SPR) induced ROS generation in AuNPs-doped ZnO hybrid under visible light. SPR-generated hot electrons flow to the CB of ZnO. (**b**) Photoexcited electrons from ZnO flow to AuNPs under UV irradiation. Reproduced from [72] with permission of Elsevier.

Noble metals, such as Ag, Au, and Pd [60,61,62,63], and transition metals (e.g., Fe, Mn, Ni and Cu) [64,65,66] can be used to dope ZnO for improving its visible-light photocatalytic activity and reducing charge carrier recombination. In the case of noble metals, the formation of a Schottky junction at the metal-ZnO interface promotes electron–hole pair separation, thereby reducing charge recombination, and enhancing spectral response in the visible region (Figure 6a) [61,67]. From this figure, the UV/visible spectrum of AuNPs-doped ZnO hybrid shows the presence of a strong band in the UV region at 300–370 nm, and a weak band in the visible region centered at around 525 nm. The small peak at ~525 nm is associated with the plasmon absorption of AuNPs dopant as evidenced by the characteristic plasmon absorption peak of colloidal AuNPs (inset). In this respect, the optical absorption of AuNPs-doped ZnO hybrid in the visible region is somewhat increased compared to that of pure ZnO. As recognized, noble metal NPs display localized surface plasmon resonance (SPR) due to the interaction of their conduction electrons with incident light. Noble metal NPs absorb light with a specific wavelength, leading to their free electrons resonate with the oscillating field of incident light. This collective electron oscillation causes a charge separation at particle surface with respect to positively charged metallic core (Figure 6b) [68]. SPR absorption induces rapid heating of metal NPs under visible light [69,70,71]. So, SPR-generated hot electrons are injected to the CB of ZnO, generating superoxide anion through a surface reaction with adsorbed oxygen, and its subsequent conversion to hydroxyl radical (Figure 7a) [72]. However, a reverse electron flow from the CB of ZnO to AuNPs occurs in AuNPs-doped ZnO hybrid under UV irradiation. The photoinduced electrons in the CB of ZnO due to UV irradiation are readily trapped by AuNPs, retarding electron–hole pair recombination accordingly. As such, ROS generation takes place on the surfaces of ZnO and AuNPs (Figure 7b).

The localized SPR intensity and wavelength depend on the factors affecting the electron charge density on nanoparticle surface, including type of particle, size, and shape. Spherical AuNPs and AgNPs exhibit strong SPR band in the visible region [73]. The wavelength of AuNPs can be tuned from visible to near infrared (NIR) light by changing their shape into nanorods. Plasmonic AuNPs find attractive applications in clinical sector for cancer therapy. AuNPs absorb incident light energy, and convert photon energy into heat. The rapid relaxation of hot electrons in targeted tissues generates localized heating capable of killing tumors, terming as plasmonic photothermal therapy (PTT) [74,75,76]. In recent years, NIR light has been used increasingly in PTT due to minimally invasive cancer treatment [73]. On the other hand, the role of AuNPs in AuNPs-doped ZnO hybrid is to inject hot electrons into the CB of ZnO for creating ROS under visible light [71,72,77,78]. The induced ROS are utilized to destroy targeted tumors. This oncological treatment is termed as photodynamic therapy (PDT). So, AuNPs act as a dual functional agent for both PTT and PDT treatments.

## 3. Fabrication of ZnO Nanostructures

**Figure 8:**
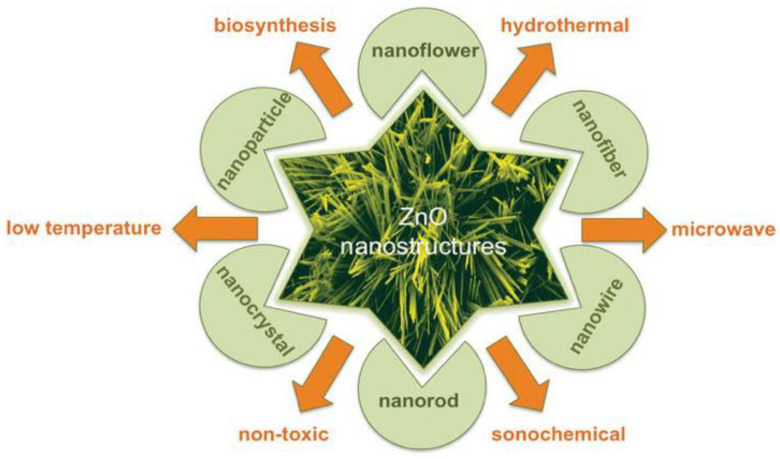
Synthetic strategies for ZnO nanostructures with various morphologies. Reproduced from [79] under the terms of the Creative Commons Attribution 3.0 license.

ZnO nanostructures with various morphologies can be synthesized using liquid-, vapor-, and solid-phase routes. Among these, liquid-phase synthesis is particularly attractive because of its simplicity, ease of fabrication, and low cost. This route is commonly used to prepare nZnO for studying their cell–material interactions in vitro in mammalian cell lines, toxic effects in animal models, and photodynamic therapy treatments for cancer cells. So, wet chemical synthesis route is briefly discussed herein. The liquid phase synthesis includes co-precipitation, hydrothermal/solvothermal, polyol and sol–gel techniques. This solution-based route gives a number of ZnO nanostructures with different sizes and shapes including spherical nanoparticles, nanorods, nanowires, tetrapods and nanoflowers (Figure 8) [79]. They can be prepared by properly monitoring the experimental parameters and conditions, such as the pressure, temperature, time, and pH, type of zinc salt, nature of solvent, etc. Most wet chemical techniques are not environmentally friendly due to the use of several harsh or harmful reagents. The co-precipitation and hydrothermal techniques generally have poor control over the size and shape of nZnO. As such, surfactants like cetyltrimethylammonium bromide (CTAB) and sodium dodecyl sulfate (SDS) are added to modify the shape and size of nZnO [80]. Triethanolamine (TEA) is also used to stabilize ZnO NPs during solvothermal synthesis [81]. However, CTAB is highly toxic, and TEA is considered as a hazardous agent towards aquatic species [82,83]. In addition, it is rather difficult to remove surfactants that are firmly attached to the synthesized nZnO surfaces. Therefore, biosynthesis of nZnO using green materials extracted from natural plants has attracted considerable attention in recent years [84,85,86,87].

### 3.1. Crystal Growth Unit (Zincate)

The co-precipitation process is most commonly used for the synthesis of nZnO. A basic solution with water/organic compound as the solvent used in dissolving metal salt precursor. The zinc salts generally employed include zinc nitrate (Zn(NO_3_)_2_), zinc chloride (ZnCl_2_), zinc sulfate (ZnSO_4_) and zinc acetate dihydrate (Zn(CH_3_COO)_2_· 2H_2_O) [88,89,90,91,92,93]. In a typical synthesis, the dissolution of zinc salt in a solvent gives Zn^2+^ ions that react with hydroxyl group to form Zn(OH)_2_ (reaction 1). By adding NaOH dropwise, Zn(OH)_2_ dissolves and reacts with OH^−^ to yield [Zn(OH)_4_]^2–^. The [Zn(OH)_4_]^2−^ (zincate) is known as the ZnO crystal growth unit (reaction 2) [88]. The Zn^2+^ and OH^−^ concentrations reach supersaturation with excessive addition of NaOH into the solution. At this stage, Zn^2+^ and OH^−^ ions form clusters in the supersaturated solution. Once a critical cluster size is reached, nucleation initiates in the solution. The nuclei then grow and coalesce into larger nanoparticles by precipitating [Zn(OH)_4_]^2−^ on positively charged Zn-terminated (0001) surface. As such, zincate ion complex is incorporated into the crystallites. These nuclei grow further along the c-axis to produce nanorods. The dehydration of [Zn(OH)_4_]^2−^ in the final stage takes place by heating the solution to a mild temperature to generate ZnO (reaction 3) [88,94]. The chemical reactions involved for the zincate formation and its subsequent dehydration to produce ZnO are given by [88,93]:

Zn^2+^ + 2OH^−^ → Zn(OH)_2_ (1)

Zn(OH)_2_ + 2OH^−^ → [Zn(OH)_4_]^2−^ (2)

[Zn(OH)_4_]^2−^ → ZnO + H_2_O + 2OH^−^ (3)

**Figure 9:**
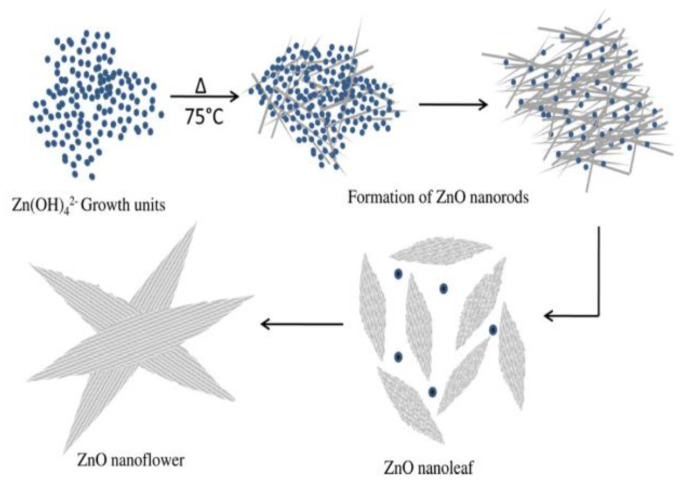
Successive stages for forming ZnO nanoflower: self-assembly of zincate to nanoparticles, nanorods, and nanoleaves. Reproduced from [92] with permission of Elsevier.

**Figure 10:**
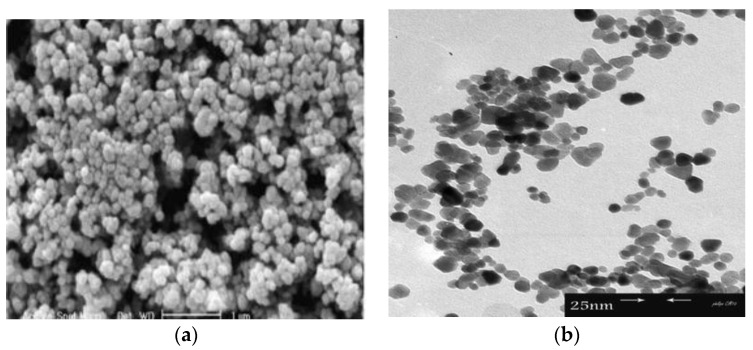
(**a**) SEM and (**b**) TEM images of ZnO nanoparticles. Reproduced from [90] under the terms of the Creative Commons Attribution 2.0 International license.

**Figure 11:**
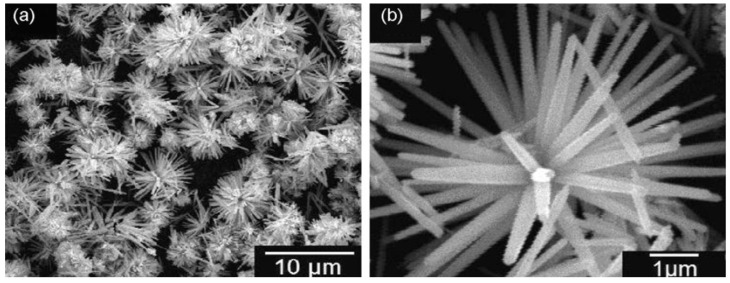
(**a**) SEM image of ZnO nanoflowers prepared by co-precipitation process. (**b**) A magnified SEM image of ZnO nanoflowers. Reproduced from [89] with permission of Elsevier.

Figure 9 shows the successive stages for precipitating ZnO nanoflower from initial zincate growth units, followed by nanoparticle and nanorod formation, and self-assembly of nanorods into leaves and nanoflower. Figure 10a,b shows typical scanning electron microscopic (SEM) and transmission electron microscope (TEM) images of ZnO NPs. Highly agglomerated nanoparticles can be readily seen in the SEM micrograph. Figure 11a,b shows the low and high magnification SEM images of ZnO nanoflowers prepared by the co-precipitation process.

**Figure 12:**
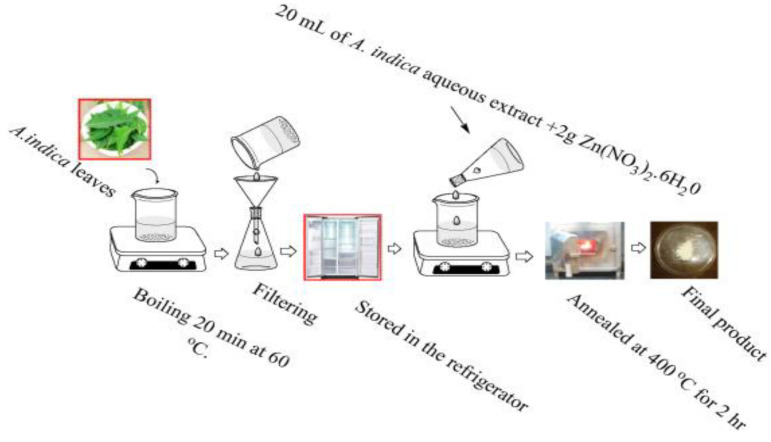
Biosynthesis of ZnO nanoparticles using Zn(NO_3_)_2_.6H_2_O and the leaf extract of *Azadirachta indica*. Reproduced from [95] with permission of Elsevier.

As mentioned, biosynthesis of ZnO nanostructures only utilizes zinc salt precursor and the plant extract without additions of harsh solvent and surfactant. Thus the synthesized products are free from hazardous residuals. The organic compounds extract from the plants are cheap and naturally abundant. Phytoextracts from the bark stems, fruits, leaves and roots of natural plants, e.g., flavonoids, phenols, coumarins, and tannins, acting both as the reducing and stabilizing agents for biosynthesis of nZnO. Those compounds react with Zn^2+^ ions through the donor-acceptor mechanism [84,85,86,87]. Figure 12 shows the cost-effective and environmentally friendly strategy for the green synthesis of ZnO nanoparticles using Zn(NO_3_)_2_.6H_2_O and the leaf extract of *Azadirachta indica* (L.) [95].

**Figure 13:**
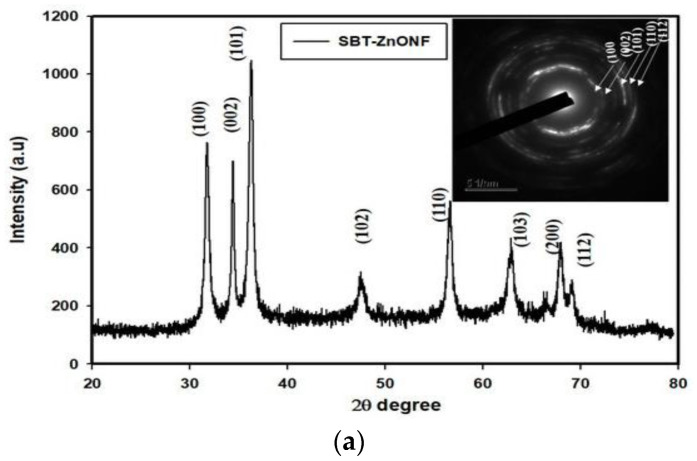

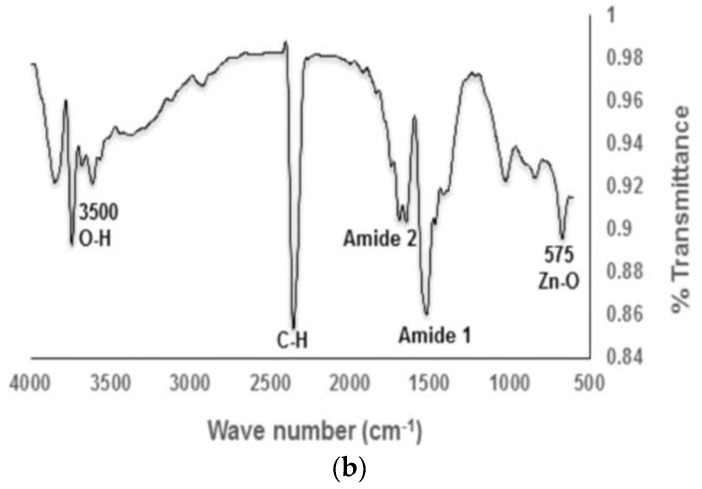
(**a**) X-ray diffraction pattern and selected area electron diffraction pattern (inset) of green ZnO nanoflowers (ZnONFs) showing the formation of a hexagonal wurtzite structure. Reproduced from [85] with permission of MDPI. (**b**) FTIR spectrum of biosynthesized ZnO NPs. Reproduced from [87] under the terms of the Creative Commons Attribution International 4.0 license.

The formation of a stable wurtzite polymorph in nZnO is necessary for inducing photocatalytic activity and offering properties to meet a wide range of applications. Several material examination techniques are typically used to characterize the structure and shape of synthesized powders. The wurtzite-type ZnO can be verified by means of XRD and selected area electron diffraction (SAED) pattern of TEM. The morphologies of nZnO are observed in the SEM and TEM. Fourier transform infrared spectroscopy (FTIR) is a powerful tool to detect the presence of Zn-O bond and other molecular moieties in the synthesized products [85,87]. Figure 13a shows a typical XRD pattern of biosynthesized ZnO nanoflowers. All the indexed diffraction peaks in the pattern confirm the wurtzite structure of ZnO nanoflowers. The electron diffraction pattern consists of a set of rings, showing nano-crystalline nature of green ZnO (inset). Figure 13b shows the FTIR spectrum of green ZnO NPs synthesized from enzyme alpha-amylase and zinc acetate dihydrate. The peak at 1650 cm^−1^ and 1540 cm^−1^ is due to N-H stretching of amide bond of proteins. The peak at 575 cm^−1^ is due to the stretching vibration of Zn with oxygen. The OH stretch of adsorbed surface water molecules appears at 3500 cm^−1^ [87].

### 3.2. Temperature Assisted Synthesis

In hydrothermal/solvothermal synthesis, water or organic solvent serves as a precipitating medium for forming nZnO. A capping agent may be added for stabilizing and restricting the growth of nanoparticles [81]. The synthesis takes place in an autoclave acting as the reaction vessel for thermal treatment. Increasing solution temperature accelerates the reactions between zinc salt precursor, solvent and capping agent as expected [81,96,97,98,99,100]. In comparison to conventional heating in hydrothermal and solvothermal processes, microwave heating offers several advantages including rapid heating, high reaction rate, and increased production yield [101,102,103,104,105,106]. These result from the ability of microwaves to react with polar molecules directly [101].

**Figure 14:**
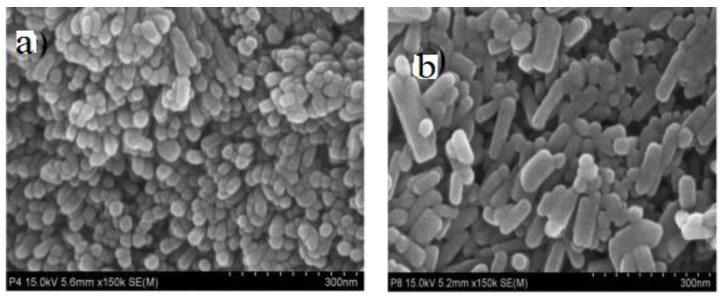
SEM images showing the morphologies of nZnO prepared from solvothermal process: (**a**) ZnO NPs in ethylene glycol for 3 h, and (**b**) ZnO nanorods in tetraethylene glycol for 3 h. Reproduced from [99] under the Creative Commons Attribution license.

**Figure 15:**
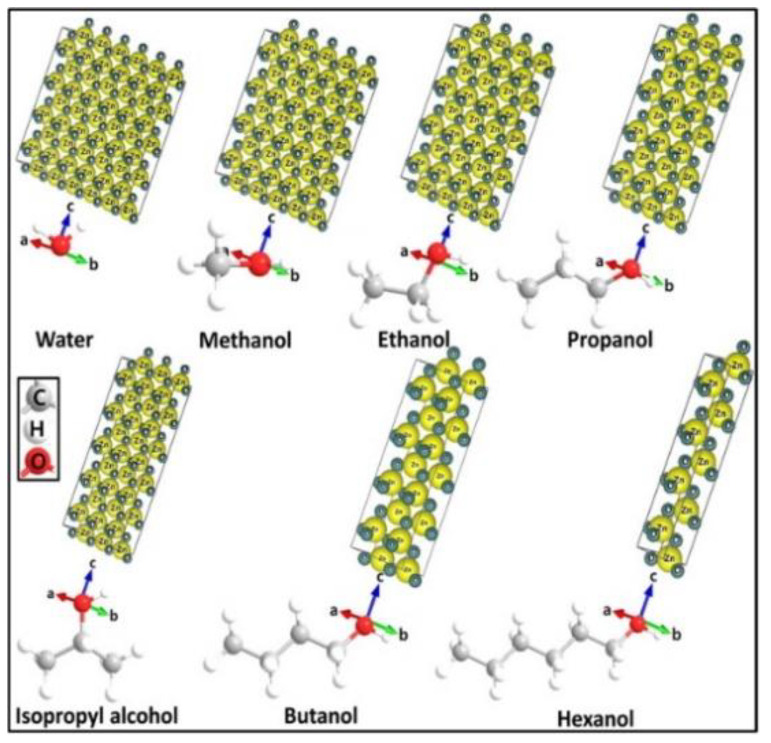
Effect of carbon chain length of alcohols on the aspect ratio of synthesized ZnO nanorods. Reproduced from [108] under the Creative Commons Attribution license.

In solvothermal synthesis, the chemical reactions take place in an organic solvent at temperatures above its boiling point. Polyols with high boiling point, high dielectric permittivity and good solubility for zinc salts are attractive solvents for preparing nZnO. Chieng and Loo synthesized nZnO by refluxing zinc acetate in ethylene glycol (EG), diethylene glycol (DEG) and tetraethylene glycol (TEG) at 160 °C for 12 h. The size of nZnO increased with increasing glycol chain length. Moreover, the shape of nZnO varied from spherical (in EG solvent), spherical and rod (DEG), to diamond-like features (TEG) [107]. Mahamuni et al. prepared nZnO by refluxing zinc acetate in DEG and TEG at 180 °C and 220 °C, respectively [99]. DEG facilitated the formation of ZnO NPs with reaction times of 2 h to 3 h; ZnO NPs with a size of ~ 15 nm were formed in DEG for 3 h (Figure 14a). ZnO nanorods were produced in TEG, and their sizes increased with increasing reflux time from 2 h to 3 h (Figure 14b). Thus the chemical structures of solvents played a key role in controlling the final size and shape of nZnO. Very recently, Ejaj et al. explained the role of hydrocarbon chain length of alkanols (solvents) on the formation of ZnO nanorods with different aspect ratios. The aspect ratios of ZnO nanorods increased linearly with the increase in carbon chain from methanol to hexanol. The long-chain carbon atoms imposed a steric hindrance and inhibited the stacking of ZnO NPs in the direction of carbon chain, thus promoting nanorod growth along the c-axis (Figure 15) [108].

### 3.3. Sol-Gel Technique

The sol–gel technique is a versatile technique for fabricating metal oxide NPs such as TiO_2_ and ZnO. The process is based on the preparation of a sol, subsequent gelation, aging and drying [109,110,111,112]. Conventionally, it involves hydrolysis and condensation reactions of metal alkoxide precursors in aqueous solutions. So, water content plays a crucial role in the sol–gel process. However, zinc alkoxides are expensive, so zinc salts (acetate, nitrate, perchlorate) are used as the precursor materials instead [113,114]. Recently, nonaqueous sol–gel process has become an attractive approach for synthesizing nZnO without water addition [111,112]. It consists of surfactant- and solvent-controlled synthesis strategies [115]. In the surfactant-controlled strategy, zinc salt is dissolved in an organic solvent, and a stabilizing agent such as monoethanolamine (MEA) or CTAB is added to the solution. For instance, Habibi and Karimi have synthesized ZnO NPs using zinc acetate, isopropyl alcohol and MEA. The mixed solution is stirred continuously on the magnetic hot plate for a certain time period until the formation of stable gel. The sol is eventually aged followed by the solvent evaporation and gel annealing [111]. In contrast, the solvent controlled strategy is relatively simple as it only needs a zinc salt and an organic solvent containing oxygen donor such as benzyl alcohol [115].

## 4. Cytotoxicity

As mentioned above, the widespread use of nZnO in cosmetic and consumer products, food additives, solar cells and medical textiles have raised concern about their toxicity to humans and animals [29,32,116,117,118,119,120,121]. The extensive utilization of nZnO results in its exposure to the human body, rendering the need for the assessment of cytotoxicity. ZnO NPs can enter human body through several paths including inhalation, skin penetration, digestive system and parenteral injection. ZnO NPs in the air easily enter human respiratory system through inhalation. Those airborne ZnO NPs come largely from industrial production plants and research laboratories relating to their synthesis, utilization and disposal processes. Occupational exposures to airborne ZnO NPs can induce toxic effects in human lungs. ZnO NPs may also induce adverse effect through the skin contact with sunscreens and antiaging creams [119]. ZnO NPs in sunscreens accumulate on the skin surface and within the skin furrows of human volunteers. ZnO NPs do not penetrate into the viable epidermis, so the penetration is largely limited to stratum corneum, a surface layer of non-viable, keratinized cells [30]. However, Gulson et al. reported that small amounts of zinc from ZnO NPs in sunscreens can penetrate through human skin and end up in blood and urine on exposure to the sun [120].

Even if ZnO NPs in sunscreens reside in stratum corneum, there remains the possibility of inducing ROS generation and releasing Zn^2+^ ions [121]. It is noted that ZnO NPs can generate excessive ROS upon exposure to UV rays of sunlight. Moreover, intrinsic oxygen vacancy defects in ZnO NPs also create ROS under visible rays of sunlight. In this respect, the possibility of ROS generation in sunscreens applied to human skins via the uptake of ZnO NPs cannot be excluded. As is known, ROS are produced in biological cells as by-products of the mitochondrial metabolism under physiological conditions. Excessive ROS induced by ZnO NPs can cause severe cellular damage. Another possibility is the release of Zn^2+^ ions in the sunscreen on the stratum corneum. Those ions may penetrate to underlying connective tissues through sweat pores and hair follicles.

**Figure 16:**
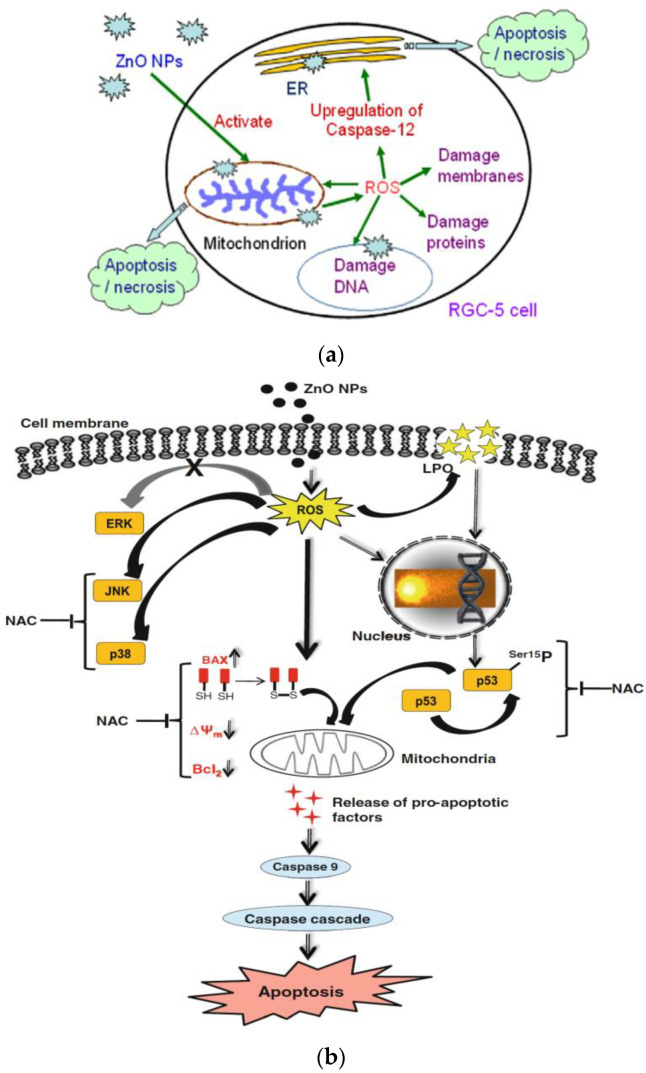
(**a**) ZnO nanoparticle-induced apoptosis/necrosis of retinal ganglion cell. Reproduced from [123] with permission of Elsevier. (**b**) Mechanisms responsible for ZnO NPs mediated toxicity and apoptosis in human liver cells. N-acetyl cysteine (NAC) is effective to inhibit Bcl2-associated X protein (Bax) induction, and p53 phosphorylation. Reproduced from [50] with permission of Springer Nature.

In general, ZnO NPs are taken-up by mammalian cells through the penetration, internalization of nanoparticles and diffusion of Zn^2+^ ions across cell membranes [50,117,122,123,124,125,126]. Nano-ZnO particles can easily penetrate cell membrane into cytoplasm, generating ROS that disrupt the functions of mitochondria, proteins and DNA molecules. The ROS production due to intracellular ZnO NPs can further induce ER oxidative stress, leading to final cell death (Figure 16a) [50,123]. The generation of ROS and oxidative stress in mammalian cells due to intracellular ZnO NPs can also induce autophagy [49]. Sharma et al. indicated that the ROS generation resulting from ZnO NPs penetration leads to a decrease in mitochondrial membrane potential (MMP; ΔΨm), upregulation of Bcl2-associated X protein (Bax) and down regulation of Bcl2 expressions, resulting in mitochondrial mediated apoptosis. Furthermore, ROS may also induce lipid peroxidation (LPO) in the cell membrane, activate p38 and JNK, damage DNA molecules in the nucleus and trigger p53 apoptotic pathway (Figure 16b) [50]. As recognized, proteins of the Bcl2 family exhibit either pro- or anti-apoptotic events. Proapoptotic proteins with multiple Bcl2 homology (BH) domains (BH1, BH2 and BH3) like Bax and Bcl2-antagonist/killer (Bak) usually oligomerize to form pores in the mitochondrial outer membrane (MOM), and induce cytochrome c release, leading to the activation of caspase 9 [127]. The tumor suppressor p53 is responsible for the transcriptional activation of proapoptotic BH3-only proteins such as Puma and Noxa. In particular, Puma (p53 upregulated modulator of apoptosis) binds to antiapoptotic Bcl2 family proteins and inhibits their activity. Thus Puma plays a role in mediating p53-induced cell death [128]. As such, Bax and Bak oligomerize readily to form pore channels in the MOM upon activation, facilitating the release of cytochrome c. So p53 is activated by phosphorylation at multiple serine (Ser) residue sites; the typical phosphorylated sites include Ser15, Ser20, Ser 33, and Ser46. The rise in p53 level in mammalian cells is generally associated with the DNA damage response.

**Figure 17:**
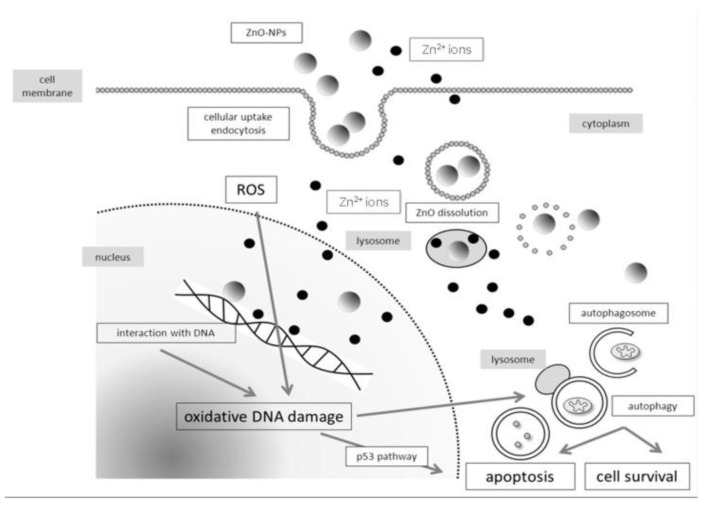
Schematic illustration showing cytotoxicity induced by zinc oxide nanoparticles, and zinc ions due to the dissolution of ZnO NPs extracellularly or intracellularly. Reproduced from [117] under the Creative Commons Attribution license.

ZnO NPs can dissolute into Zn^2+^ ions extracellularly. Such Zn^2+^ ions can penetrate cell membrane into cytoplasm, inducing the generation of ROS and oxidative stress in a wide range of cell types [122]. Moreover, ZnO NPs are also internalized by endocytosis, and directed into the acidic environment of lysosomes, leading to their degradation into Zn^2+^ ions [124,125,126]. Very recently, Zhang et al. demonstrated that ZnO NPs can induce autophagy, thereby enhancing the dissolution of ZnO NPs in lysosomes for releasing Zn^2+^ ions [125]. The cytotoxicity derives from the cellular uptake of ZnO NPs and extracellular Zn^2+^ ions, and intracellular Zn^2+^ ions due to lysosomal dissolution is depicted in Figure 17 [117].

The intracellular Zn^2+^ ions also induce ROS, resulting in oxidative DNA damage and apoptosis [126]. From the literature, ROS trigger mitogen-activated protein kinases (MAPKs), including p38 kinase, extracellular signal-regulated kinase (ERK), and c-Jun amino-terminal kinase (JNK) in human lymphocyte (Jurkat) cells [116]. In this respect, JNK and p38 MAPK are activated upon phosphorylation in response to ROS production. The activation of p38MAPK and JNK has been related to apoptotic cell death [129]. The ROS production can further induce ER stress [33,35,123]. Severe ER stress leads to the activation of JNK and C/EBP homologous protein (CHOP), resulting in the disruption of protein folding mechanism [130]. ER is an important organelle needed for the maintenance of intercellular Ca^2+^ homeostasis, and for the synthesis, folding as well as transport of proteins. In addition, ER communicates with mitochondria via mitochondria-associated ER membranes (MAMs), acting as a site for regulating various complicated signal pathways for Ca^2+^ homeostasis and apoptosis [131].

### 4.1. Cell Cultivation

#### 4.1.1. Normal Cells

ZnO nanostructures can induce cytotoxic and genotoxic effects to normal mammalian cells [132,133,134,135,136,137,138,139,140,141,142,143,144,145,146] and cancerous cells [50,54,147,148,149,150,151,152,153,154,155,156,157,158,159,160,161,162]. For normal human bronchial epithelial (BEAS-2B) cells, cellular uptake of ZnO NPs leads to their dissolution into intracellular Zn^2+^ ions [135]. The toxic effects of nZnO in normal cells depends greatly on their size, shape, and concentration, as well as culture time and cell type. In general, nZnO exhibits concentration-, size-, and shape-dependent toxicity towards different cell lines. Khan et al. synthesized Zn nanorods with sizes of 47.8–52.5 nm using sol–gel process, and studied their toxic effect in human erythrocytes (red blood cells; RBCs) [140]. ZnO nanorods induced a dose dependent hemolytic activity to RBCs as a result of ROS production. Due to the lack of nuclei and mitochondria, RBCs have a poor repair capacity after an injury. So, ZnO NPs disrupted the integrity of cell membranes of RBCs through LPO, leading to the membrane damage and hemolysis. Moreover, ZnO nanorods also induced DNA damage through the ROS generation. Gu et al. demonstrated that ZnO NPs induce cytotoxicity in HUVECs due to intracellular ROS generation. The dose-dependent cytotoxicity was caused by the accumulation of intracellular Zn^2+^ ions [142].

Keratinocyte is the main cell type in the epidermis. ZnO NPs disrupt mitochondria function of human epidermal keratinocytes (HaCaT), and promote membrane leakage of LDH upon exposure to 0, 10, 20, 40, and 80 μg/mL for 24 h. These derive from the induction of oxidative stress due to the ROS generation and LPO [136]. Kocbek et al. exposed human keratinocytes to commercial ZnO nanorods for short-term (24 h, 48 h and 72 h) with doses of 0.5, 10, 15, 20, 25, 50, and 100 μg/mL, and long-term (three months) at doses of 0.5, 5, and 10 μg/mL [133]. For short-term exposures, ZnO nanorods reduced cell viability at doses ≥ 15 μg/mL. Prolonged exposure to ZnO NPs at 10 μg/mL for three months led to a decrease in mitochondrial activity owing to the dissolution of ZnO in the cell culture medium, and Zn^2+^ ion-induced ROS generation. Very recently, Wang et al. investigated cytotoxic effects of ZnO NPs (55 nm) with doses of 10, 15, 30, and 100 μg/mL on different cell types including HaCaT, human gingival fibroblast (HGF-1), and human gingival squamous carcinoma cell line (Ca9-22) [135]. ZnO-NPs were less toxic to normal HaCaT and HGF-1 cells, but showed severe toxicity to Ca9-22 cells at doses ≥ 30 μg/mL. Thus, ZnO NPs exhibited selective toxicity to cancer cells due to the ROS generation, mitochondrial oxidative damage, and DNA fragmentation. Yu et al. synthesized ZnO nanorods [15.38 nm (width), 82.34 nm (length)] in in a basic alcohol solution using co-precipitation process. They then assessed the toxic effect induced by ZnO nanorods at doses of 5, 10 and 20 µg/mL on murine skin epidermal normal cells (JB6 Cl 41-5a). After cellular uptake, ZnO NPs induced ROS in a dose- and time-dependent manner. This led to abnormal autophagic vacuoles accumulation and mitochondria dysfunction [137].

**Figure 18:**
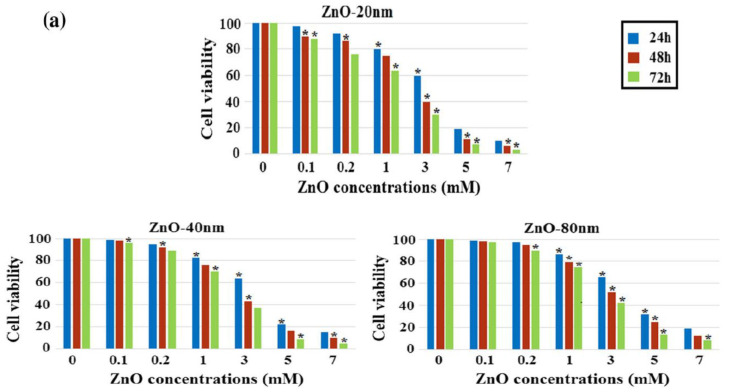

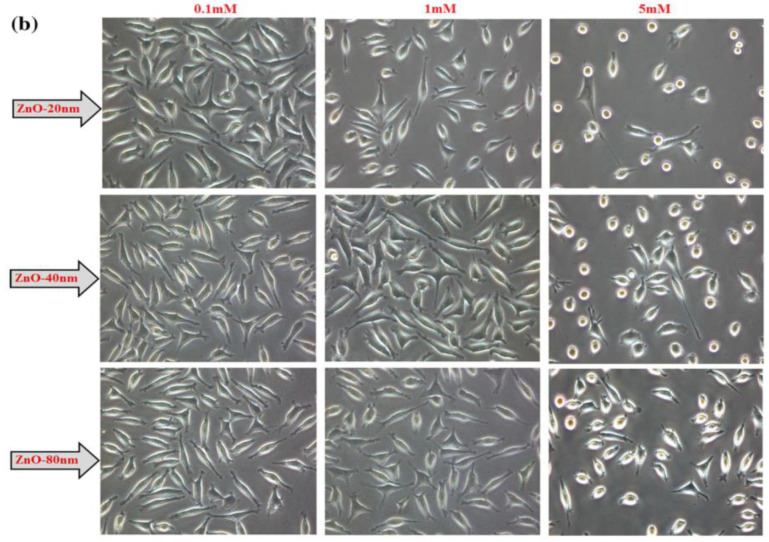
(**a**) Viability of L929 murine fibroblasts treated with nZnO of different sizes and doses for 24, 48 and 72 h. The results are presented as mean ± standard deviation of three independent experiments. * *p* < 0.05. (**b**) Optical images showing the morphologies of L929 cells exposed to nZnO of different sizes and doses for 48 h. Images are taken with x20; arrow scale bar: 20 µm. Reproduced from [144] with permission of Springer Nature.

Meyer et al. treated human dermal fibroblasts with commercial ZnO NPs (20 nm) at doses of 2.5, 5, 25, 50, and 100 μg/mL. The cell viability fell to below 15% at ZnO NPs doses ≥ 25 μg/mL as determined by 3-(4,5-dimethylthiazol-2-yl)-2,5-diphenyltetrazolium bromide (MTT) assay. As such, ZnO NPs markedly induced cell death through the upregulation of p53 and p38 pathways. The p38 MAPK phosphorylated p53 at Ser33 and Ser46. Increased Ser33 and Ser46 phosphorylation of p53 induced apoptotic target gene transcription [134]. Papavlassopoulos demonstrated that spherical ZnO NPs (∼150 nm) were more toxic than ZnO nanotetrapods in normal human dermal fibroblasts (NHDF) [53]. Spherical ZnO NPs with a larger surface area released more Zn^2+^ ions than tetrapods for inducing cytotoxicity. Recently, Sirelkhatim et al. investigated the effect of particle size and shape of nZnO on the viability of mouse fibroblast cell line (L929) [144]. ZnO nanorods with the widths of 80 nm and 40 nm, and nanoparticles (20 n) were used in their study. From the 3-(4,5-dimethylthiazol-2-yl)-5-(3-carboxymethoxyphenyl)-2-(4-sulfophenyl)-2H-tetrazolium (MTS) assay, cytotoxicity induced by ZnO NPs was size- and dose-dependent, especially for the cells treated with smallest ZnO NPs (20 nm) (Figure 18a). The morphologies of L929 cells treated with nZnO of different sizes and doses for 48 h were depicted in Figure 18b. No morphological alterations were observed for the cells exposed to low and medium doses of nZnO (0.1 and 1 mM) of various sizes. However, cell viability reduced markedly at a high dose of 5 mM. The cells changed their shape to round features. These rounded cells reflected the early process of apoptosis. The apoptotic cell appeared as a round feature due to cell shrinkage.

Because of their small sizes, ZnO NPs are internalized readily by immune cells such as monocytes, macrophages, and dendritic cells. Song et al. have conducted an earlier study on the cytotoxicity of commercial nZnO and micro-ZnO (fine ZnO) to murine macrophages (Ana-1) [132]. ZnO nanorods of different sizes (width: 100 nm, length: 107.6 nm; width: 30 nm, length: 70.89 nm), fine ZnO rods (width: 173.48 nm, length: 341.75 nm), and spherical ZnO nanoparticles (10–30 nm) are employed in their study. A dose-dependent cytotoxicity is observed for fine ZnO rods and nano-ZnO as revealed by the cell viability, lactate dehydrogenase (LDH) and ROS level measurements. In particular, spherical ZnO NPs (10–30 nm) exhibit the highest toxicity comparing with ZnO nanorods. Such nanoparticles trigger a higher production of ROS than fine ZnO rods due to their large surface area and high surface reactivity. The cytotoxicity of ZnO nanorods and ZnO NPs derives from the Zn^2+^ ions released into the culture media as evidenced by inductively coupled plasma atomic emission spectroscopy (ICP-AES). Those Zn^2+^ ions then induce the ROS generation and the leakage of LDH from the cell membrane. Recently, Johnson et al. reported that the exposure of immune cells to ZnO NPs results in autophagy and excessive intracellular ROS production. Released Zn^2+^ ions from ZnO NPs are taken up by the cells, thereby triggering excessive generation of intracellular ROS and autophagic death of immune cells [142]. Roy et al. studied cytotoxic effect of commercial ZnO NPs (∼50 nm) on mouse primary peritoneal macrophages. They reported that ZnO NPs induce ROS generation and promote lipid peroxidation in macrophages. These lead to the autophagy activation, resulting in apoptosis as revealed by the cleavage of apoptosis markers such as caspases 3, 8, and 9 [138].

**Figure 19:**
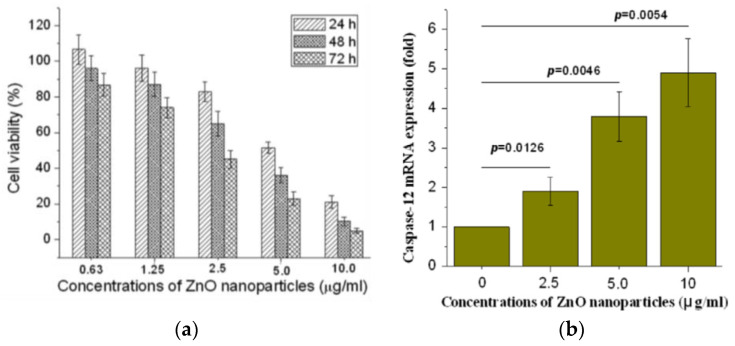
(**a**) Cell viability of RGC-5 cells vs ZnO NPs concentrations. The results are presented as mean ± standard deviation of three independent experiments (n = 3). (**b**) Real-time quantitative PCR analysis of caspase-12 gene expression in RGC-5 cell treated with ZnO NPs concentrations of 2.5, 5.0 and 10.0 µg/mL. The results are expressed as mean ± S. E. of three independent experiments. Reproduced from [123] with permission of Elsevier.

Guo et al. exposed murine retinal ganglion cells (RGC-5) to ZnO NPs (60 nm). MTT assay was used to assess the cytotoxicity of nanoparticles [123]. A dose-dependent effect of ZnO NPs on cell viability was produced (Figure 19a). The half maximal inhibitory concentration (IC_50_) values of ZnO NPs on RGC-5 cells were 5.19, 3.42, and 2.11 µg/mL for 24, 48, and 72 h, respectively. ZnO NPs treatment led to a reduction of mitochondria potential and excessive generation of ROS (i.e., hydrogen peroxide and hydroxyl radical) levels in RGC-5 cells. Consequently, caspase 12 protein was activated, triggering an endoplasmic reticulum (ER)-specific apoptosis pathway and cellular damage as shown in Figure 16a. Moreover, high levels of caspase 12 were induced by ZnO NPs in a dose-dependent manner (Figure 19b).

The translocation of inhaled metal and metal oxide nanoparticles through the olfactory bulb–brain pathway could lead to neurotoxicity by activating astrocytes and microglia in the brain. The deposition of such nanoparticles in the brain would trigger oxidative stress and apoptosis. Wang et al. exposed mouse primary astrocytes to ZnO nanorods (average size of 45 nm) at doses of 4, 8 and 12 µg/mL for 6, 12, and 24 h. They found that ZnO nanorods reduce cell viability, increase LDH and ROS levels, and activate caspase-3 in a dose- and time-dependent manner [139]. Deng et al. reported that ZnO NPs reduce viability of neural stem cells (NSCs) in a dose-, but not size-dependent manner. Commercial ZnO NPs of different sizes, i.e., 10, 30 and 60 nm at doses of 3, 6, 12, 18, and 24 ppm were used in the study [145]. Exposure of NSCs to ZnO NPs of all sizes at doses ≥ 12 ppm for 24 h led to apoptosis due to the dissolved Zn^2+^ ions in the culture medium or inside cells. No toxic effects were observed for NSCs treated with ZnO NPs of all sizes at doses ≤ 6 ppm for 24 h.

**Figure 20:**
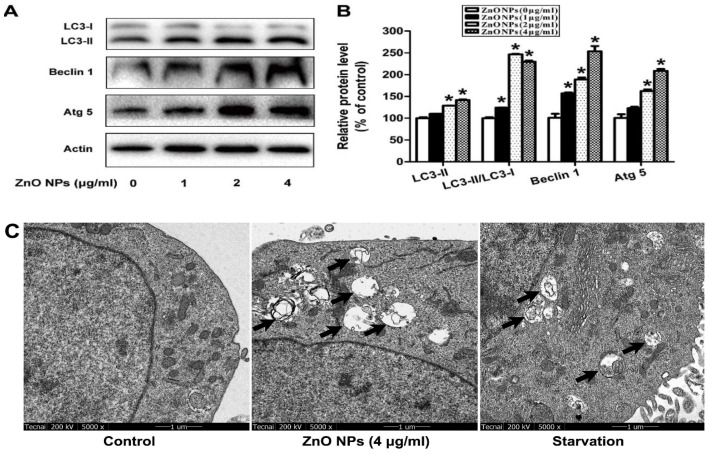
ZnO NPs induces autophagy of GC-1 spg cells: (**a**) Western blot analysis of protein levels of ATG5, Beclin1 and LC3 in the cells treated with 4 μg/mL ZnO NPs for 24 h. Actin is employed as an internal control. (**b**) Densitometry quantification of relative protein levels. (**c**) TEM images showing autophagic vacuoles of untreated cells (control), and cells treated with 4 μg/mL ZnO NPs for 24 h. Starvation-treated cells act as a positive control. Autophagic vacuoles are indicated by the arrows. * *p* < 0.05 compared to control. Reproduced from [49] under the Creative Commons Attribution license.

**Figure 21:**
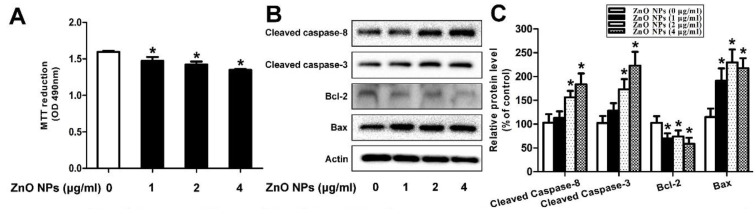
ZnO NPs induces apoptosis in murine GC-1 spg cells treated with 1–4 μg/mL ZnO NPs for 24 h. (**a**) Cell viability determined from 3-(4,5-dimethylthiazol-2-yl)-2,5-diphenyltetrazolium bromide (MTT) assay, (**b**) protein levels of Bax, Bcl2, cleaved caspase 3 and cleaved caspase 8, and (**c**) relative protein level vs control. * *p* < 0.05 compared to control. Reproduced from [49] under the Creative Commons Attribution license.

In the case of reproductive system, ZnO NPs induced significant cytotoxicity, apoptosis, and autophagy in murine Leydic, Sertoli and GC-1 spg cells [49,143,146]. Shen et al. reported that ZnO NPs (30 nm) had adverse effects in Leydic cells by activating autophagy and apoptosis via oxidative stress generation [146]. Yang et al. indicated that ZnO NPs also induced autophagy and apoptosis in mouse-derived spermatogonial cells (GC-1 spg) due to the generation of ROS and induction of oxidative stress. The malondialdehyde (MDA) level in the ZnO NPs-treated cells was markedly increased, while the GSH and antioxidant superoxide dismutase (SOD) levels were decreased significantly, showing the oxidative effect [49]. Thus, autophagy was activated during stress conditions, facilitating the degradation of cellular components by the vacuoles or lysosomes. As recognized, autophagy was a self-degradative process of cellular components for maintaining homeostasis. However, the production of excessive intracellular ROS would lead to autophagic death of GC-1 spg cells. The autophagy proteins such as microtubule-associated protein 1A/1B light chain 3 (LC3), autophagy related 5 (ATG5) and Beclin 1 were elevated as revealed by Western blot analysis and densitometry quantification (Figure 20a,b). LC3 generally appeared in two forms, i.e., LC3-I and LC3-II. The former was cytosolic, while the latter was membrane-bound. LC3-II was distributed on isolation membranes of autophagosomes. The LC3-II level was closely correlated with the number of autophagosomes, serving as a biomarker for autophagosome formation [163]. Apparently, treatment with ZnO-NPs at doses ≥ 2 µg/mL induced production of high LC3-II, Beclin1 and ATG5 levels. TEM micrographs showed that untreated cells had few autophagosomes in the cytoplasm. However, a marked increase of autophagic vacuoles was observed in ZnO NPs-treated cells, containing degraded organelles, such as mitochondria and endoplasmic reticulum (Figure 20c). Moreover, ZnO NPs inhibited cell viability in a dose-dependent manner. ZnO NPs with doses ≥ 2 µg/mL upregulated the protein levels of caspase 8, caspase 3, and Bax, and downregulated Bcl2 level, leading to apoptosis (Figure 21a–c).

**Figure 22:**
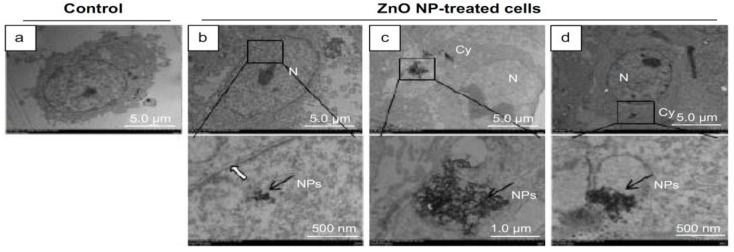
Transmission electron microscopic images showing (**a**) untreated Leydic cell (control), and (**b**–**d**) localization of ZnO NPs within the nucleus (N) and cytoplasm (Cy) of the cell. Bottom panel: high magnification views of the framed region. Reproduced from [143] with under the Creative Commons Attribution—Non Commercial (unported, v3.0) license.

**Figure 23:**
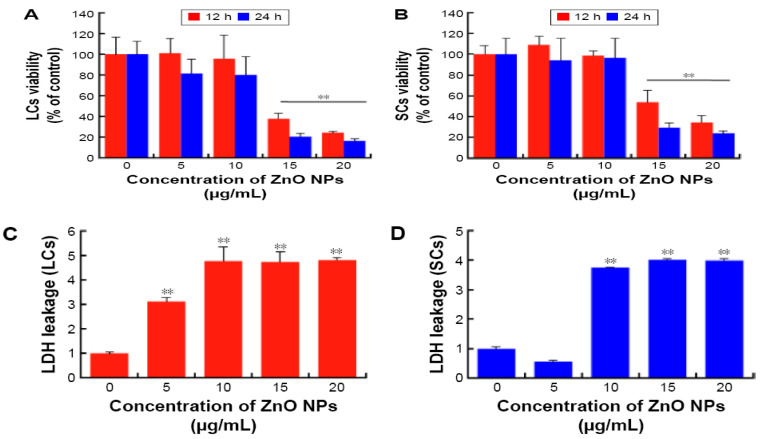
Viability of (**a**) Leydic cells and (**b**) Sertoli cells treated with ZnO NPs of various concentrations for 12 and 24 h as determined with MTT cell proliferation assay. LDH leakage level of (**c**) Leydic cells and (**d**) Sertoli cells. Data are presented as mean ± standard deviation of three separate experiments, in triplicate. ** *p* < 0.01. Reproduced from [143] under the Creative Commons Attribution—Non Commercial (unported, v3.0) license.

**Figure 24:**
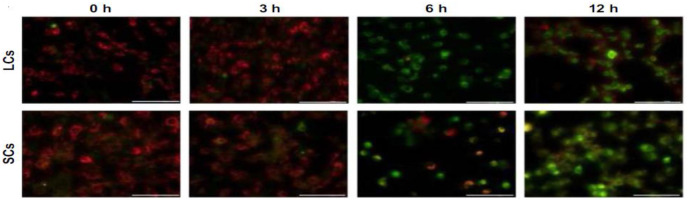
Changes in mitochondrial membrane potential of Leydic cells (LCs; top panel) and Sertoli cells (SCs; bottom panel) treated with 15 µg/mL ZnO NPs for 0, 3, 6, and 12 h using cationic JC-1 dye. Reproduced from [143] under the Creative Commons Attribution.

Han et al. submitted Leydic cells and Sertoli cells to spherical ZnO NPs with sizes ranging from 20 to 110 nm at doses of 5, 10, 15, and 20 µg/mL for 12 h and 24 h. Both types of cells internalized ZnO NPs, resulting in a time- and dose-dependent toxicity and final cell death [143]. Figure 22a–d are TEM micrographs showing internalization of ZnO NPs by Leydig cells. The MTT assay results reveal that the viability of Leydig cells and Sertoli cells drop significantly at ZnO NPs doses ≥ 15 µg/mL for 12 h and 24 h exposures. In other words, the viability of Leydic cells and Sertoli cells falls to about 20% and 28% respectively after treating with ZnO NPs for 24 h (Figure 23a,b). This is accompanied by a surge in LDH leakage level (Figure 23c,d), increased ROS level, and loss of mitochondrial membrane potential (Figure 24). In general, fluorescent cationic JC-1 dye is typically used as an indicator for monitoring mitochondrial membrane potential (MMP) of a cell. The positively-charged dye penetrates healthy cells and accumulates in the negatively charged mitochondria with a high MMP. The dye forms J-aggregates that emit red fluorescence at a critical concentration. However, the dye penetrates easily through the mitochondrial membranes of apoptotic cells. In this case, the accumulation and formation of J-aggregates are inhibited, so green fluorescence of original dye is observed for the dead cells with collapsed MMP [164].

#### 4.1.2. Cancerous Cells

**Figure 25:**
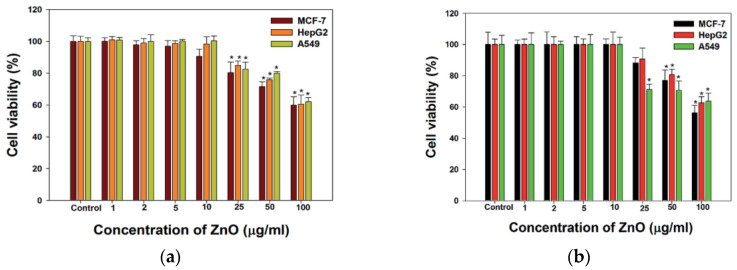

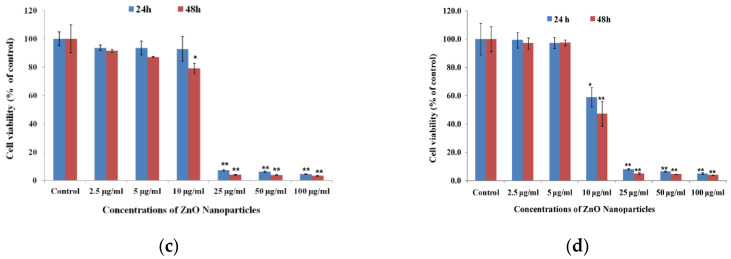
The viability of MCF-7, HepG2 and A549 cells treated with ZnO NPs of different doses for 24 h as determined by (**a**) MTT and (**b**) neutral red uptake (NRU) assays. Error bars indicate standard deviation of three independent experiments (n = 3). * *p* < 0.05, ** *p* < 0.001 versus control. Reproduced from [157] with permission of the Royal Society of Chemistry under the Creative Commons Attribution license. MTT cell viability results of (**c**) MCF-7 and (d) HepG2 cells exposed to ZnO NPs of various doses for 24 and 48 h. The values are expressed as mean ± SD of three independent experiments. * *p* < 0.01 and ** *p* < 0.001 vs. control group. Reproduced from [150] with permission of Elsevier.

**Figure 26:**
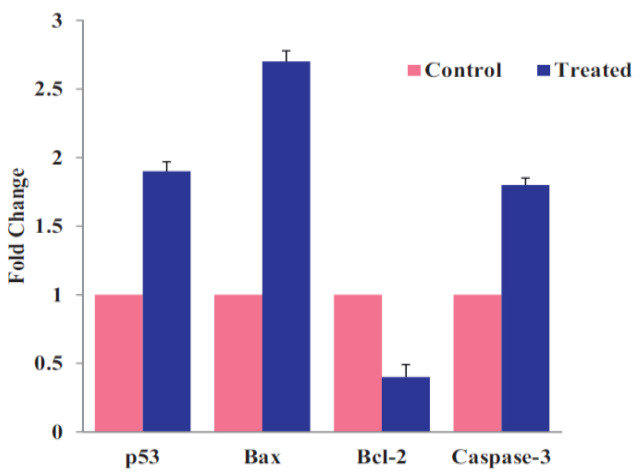
The mRNA expression levels of apoptotic markers in HepG2 cells treated with ZnO NPs at a dose of 50 µg/mL for 24 h. The data are presented as mean ± standard deviation of three identical experiments with three replicates. Reproduced from [150] with permission of Elsevier.

Nano-ZnO materials have been reported to attack multiple cancer cell lines based on cytotoxic assay results [50,126,147,148,149,150,151,152,153,154,155,156,157,158,159,160,161,162]. Very recently, Hussain et al. synthesized spherical ZnO NPs (90 nm) from Pandanus odorifer leaf extract and zinc acetate dihydrate. The anticancer activity of green ZnO NPs of different concentrations (1, 2, 5, 10, 25, 50, and 100 µg/mL) against MCF-7 (breast cancer), HepG2 (liver cancer), and A549 (human lung adenocarcinoma) cells using MTT and neutral red uptake (NRU) assays was studied [157]. The anticancer activity of green ZnO NPs to human carcinoma cells was found to be dose-dependent. Green ZnO NPs inhibited the proliferation of cancer cells at doses ≥ 50 μg/mL. At 50 and 100 μg/mL, the viability of all cancer cells decreased to only 70% and 60%, respectively (Figure 25a). Similar viability results for these cancer cells were obtained using NRU assay (Figure 25b). Wahab et al. also exposed MCF-7 and HepG2 cells to spherical ZnO NPs (13 nm) of different concentrations prepared by co-precipitation process. ZnO-NPs induced cytotoxicity and apoptosis in MCF-7 and HepG2 cancer cells at doses ≥ 25 μg/mL (Figure 25c,d). Comparing to green ZnO NPs (90 nm), smaller ZnO NPs (13 nm) acted more destructive to MCF-7 and HepG2 cells as expected. The apoptotic cell death derived from the ROS and oxidative stress induction through upregulation of the mRNA expression levels of Bax, p53 and caspase 3, and downregulation of anti-apoptotic Bcl2 gene (Figure 26) [150]. Moghaddam et al. treated MCF-7 cells with biosynthesized ZnO NPs (10–59 nm) at doses of 62.5, 125, 250, 500, and 1000 µg/mL. ZnO NPs arrested the cell cycle in the G2/M phase, upregulated pro-apoptotic genes p53, p21, Bax, and JNK, and downregulated anti-apoptotic genes Bcl2, AKT1, and ERK1/2 in a dose-dependent manner [152]. Akhtar et al. studied the cytotoxicity of ZnO NPs (21.34 nm) prepared by the co-precipitation process against HepG2 and A549 cancer cells as well as normal primary rat cells (astrocytes and hepatocytes) [148]. ZnO NPs exhibited a dose-dependent cytotoxic effect in HepG2 and A549 cancer cells. The cytotoxicity was not found in normal rat cells. In particular, apoptotic protein levels of p53 and Bax were highly upregulated while the Bcl2 level was markedly downregulated in ZnO NPs treated cells [148]. Heim et al. reported a dose- and time-dependent toxicity of ZnO NPs (15–18 nm) in A549 cells. ZnO NPs first dissolved into Zn^2+^ extracellularly, then the uptake of Zn^2+^ induced ROS and DNA-double strand breaks [122].

In other cancer cell lines, ZnO spherical particles (26 nm) and ZnO nanorods (62 and 90 nm) induced size-, dose-, and time-dependent cytotoxicity in human colon carcinoma (Caco-2) cells. These led to the generation of ROS and oxidative stress, and LDH leakage accordingly [149]. A recent study by Liu et al. showed that commercial ZnO NPs of different sizes (average size of 18.5 nm and 47.1 nm) would induce neurotoxicity in human neuroblastoma SHSY5Y cells in a size- and time-dependent manner [154]. The cells were treated with ZnO NPs at 5, 10, 20, 40, 60, 80, and 100 µg/mL for 2, 6, 12, and 24 h. Toxic effects were more significant at concentrations ≥ 20 μg/mL, especially for the cells exposed to smaller ZnO NPs (18.5 nm) for time periods ≥ 12 h. Intracellular Zn^2+^ ions due to the dissolution of ZnO NPs in lysosome induced the generation of ROS and oxidative stress, leading to mitochondria dysfunction, cytoskeletal disruption, and apoptosis. Wang et al. observed a decline in the viability of mouse glioblastoma and neuroblastoma cell line by treating with ZnO nanowire arrays [155]. From the literature, ZnO NPs (20 nm) with doses of 5, 10, 20, and 30 µg/mL induced dose- and time-dependent toxicity in human ovarian cancer cells (SKOV3). Apoptosis appeared at doses ≥ 20 μg/mL through the generation of ROS, reduction of MMP, upregulation of p53, LC3, Bax, caspase 9, and downregulation of Bcl2 [156].

**Figure 27:**
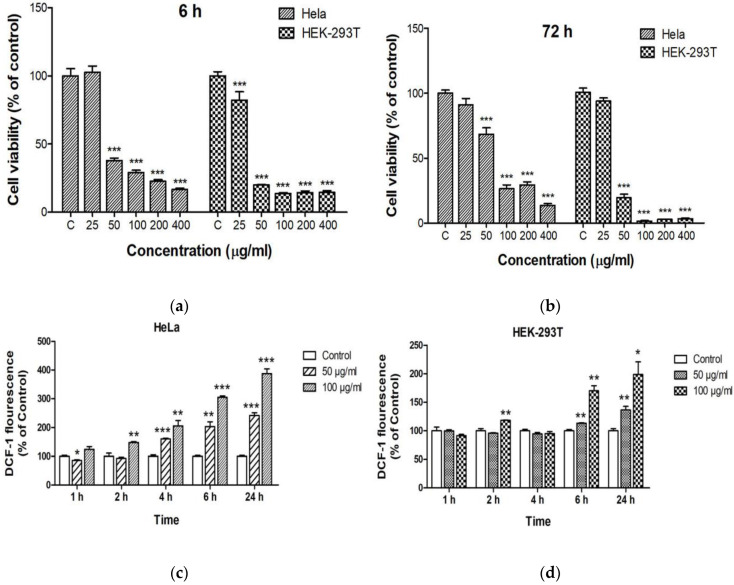
CCK-8 assay cell viability results for HeLa and HEK-293T cells treated with ZnO QDs at different concentrations for (**a**) 6 h and (**b**) 72 h. All data are presented as mean ± SD (n = 3). *** *p* < 0.001 versus control (C). ROS generation in (**c**) HeLa and (**d**) HEK-293T cells treated with ZnO QDs at doses of 50 and 100 µg/mL for 1, 2, 4, 6, and 24 h. Dichlorofluorescein (DCF) fluorescence intensity is normalized relative to control. All data are presented as mean ± SD (n = 3). * *p* < 0.05, ** *p* < 0.01 and *** *p* < 0.001 versus control. Reproduced from [162] under the terms of the Creative Commons Attribution license.

Reshma and Mohanan reported that spherical ZnO NPs (25–40 nm) with doses of 5, 15, 25, 50, 75, and 100 µg/mL induced toxicity in human embryonic kidney (HEK 293) cells in dose and time dependent manner. This was attributed to the formation of ROS and the induction of oxidative stress, leading to the loss of mitochondrial potential and lysosomal activity [147]. HEK 293 and its derivatives had been used widely in cell biology after human cervix epithelial adenocarcinoma (HeLa) cells. Very recently, Yang et al. treated HEK 293T and HeLa cells with zero-dimensional ZnO quantum dots (QDs; 7.10 nm) at doses of 25, 50, 100, 200, and 400 µg/mL for 6, 24, 48, and 72 h. ZnO QDs induced obvious cytotoxicity at a dose of 50 µg/mL. HEK-293T cells showed lower viability than HeLa cells at doses ≥ 50 µg/mL (Figure 27a,b) [162]. Furthermore, ZnO QDs also induced ROS generation in both cell types in a time and dose-dependent manner (Figure 27c,d). Altogether, these led to a loss of MMP and eventual cell death or apoptosis.

As previously mentioned, ZnO NPs exhibited preferential cytotoxicity against HepGe2 cells [148]. Several studies in the literature reported the selective killing of nZnO against cancerous cells without UV/visible light irradiation [135,144,148,159,160,161]. An earlier study by Hanley et al. showed that ZnO NPs (8–13 nm) induced a preferential killing of Jurkat leukemic and Hut-78 lymphoma T cell lines with 28–35 times sensitivity relative to normal T cells [161]. Cancerous T cells produced a higher ROS level than normal T cells. The elevated production of ROS in cancerous T cells caused oxidative damage to cellular molecules such as lipids, proteins, and DNA, and induced organelle dysfunction. The oxidative degradation of lipids resulted in the creation of lipid peroxidation, leading to the initiation of chain reactions and subsequent disruption of organelle and cell membranes.

**Figure 28:**
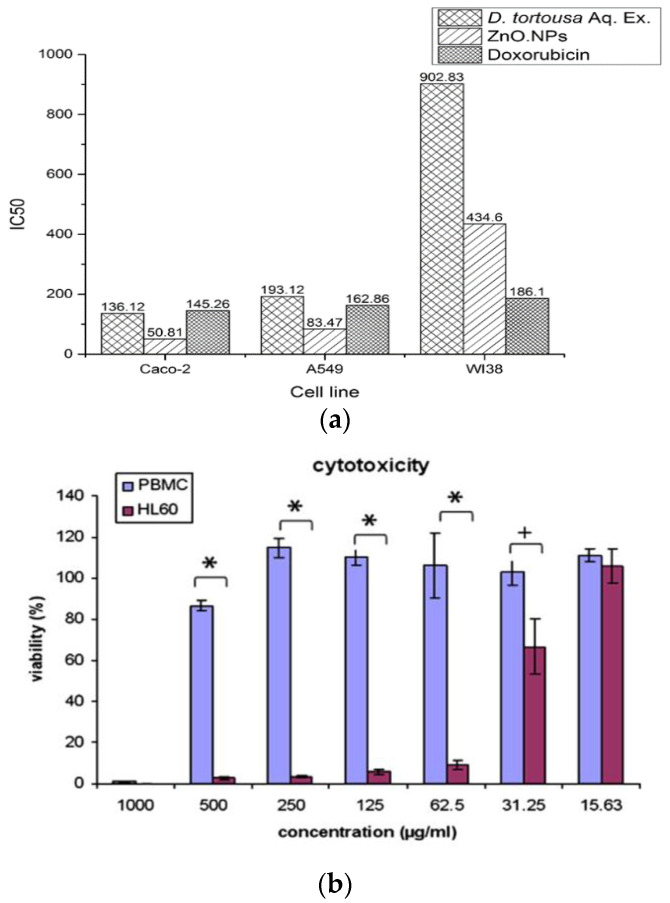
(**a**) The half maximal inhibitory concentration (IC50; µg/mL) for Deverra tortuosa aqueous extract, green ZnO NPs and doxorubicin on Caco-2 and A549 cancer cells as well as WI38 normal cells. IC50: the concentration of tested agent with 50% inhibition of the cell viability. Doxorubicin: chemotherapy drug for treating cancer cells. Reproduced from [159] with permission of Springer Nature under a Creative Commons Attribution 4.0 International License. (**b**) Selective cytotoxic effects of ZnO NPs on HL60 cancer cells and normal PBMCs. The data are expressed as mean ± standard deviation of three identical experiments with three replicates. * *p* < 0.0001, and ^+^ *p* < 0.05. Reproduced from [160] with permission of Elsevier.

**Figure 29:**
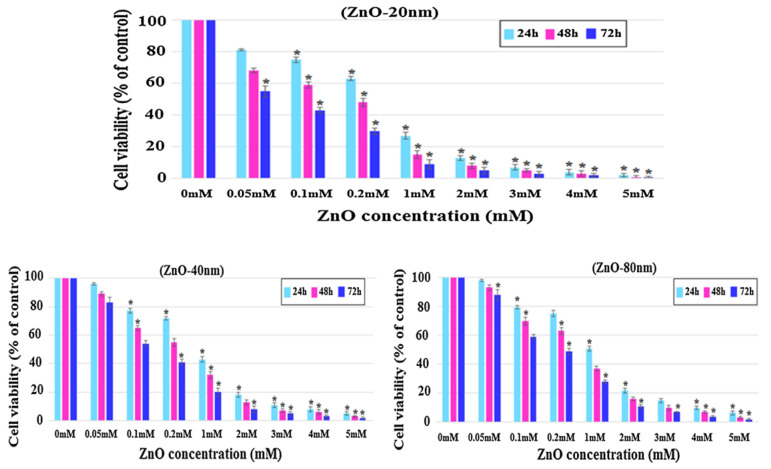
Cell viability of HeLa cancer cells treated with ZnO NPs of different sizes and doses for 24, 48 and 72 h. Results are presented as mean ± standard deviation of three independent experiments. * *p* < 0.05. Reproduced from [144] with permission of Springer Nature.

Selim et al. demonstrated that green ZnO NPs (9.26–31.18 nm) synthesized from Deverra tortuosa extract selectively induce apoptosis in Caco-2 and A549 cells rather than in human lung fibroblast cell line (WI38). Moreover, Caco-2 cells were more sensitive than A549 upon exposure to green ZnO NPs (Figure 28a) [159]. Premanathan et al. reported that ZnO NPs exhibited a preferential ability to kill human myeloblastic leukemia cells (HL60) as compared with normal peripheral blood mononuclear cells (PBMCs) [160] (Figure 28b). Comparing to viability of normal L929 cells (Figure 18a), ZnO nanomaterials induced selective killing towards HeLa cells as shown in Figure 29. Cellular apoptosis in HeLa derived from the generation of ROS and released Zn^2+^ ions [144]. Apparently, the selective killing of various cancerous cells by ZnO NPs opens the opportunities for chemists and materials scientists in the development of therapeutic agents for malignant tumors. Table 1 lists the cytotoxic effects induced by ZnO nanostructures to normal mammalian cells and cancer cells.

### 4.2. Photocatalytic Toxicity

**Figure 30:**
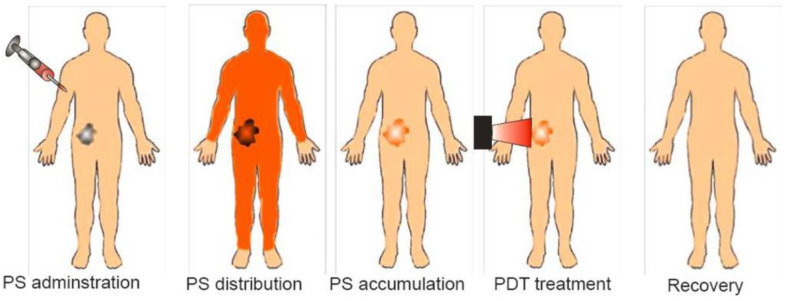
Schematic showing photodynamic therapy (PDT) by injecting a photosensitizer (PS) into human body. PS is then distributed and accumulated in tumor tissues. This is followed by ultraviolet light irradiation to generate ROS for killing cancer cells. Reproduced from [171] under the Creative Commons Attribution license.

Generally, chemotherapy and radiotherapy are commonly used in the clinical sector to treat cancer cells. However, these therapy treatments often introduce many serious side effects. As mentioned, ZnO nanostructures exhibit cytotoxic effect against several cancer cell lines. In this respect, they show great potential for use in cancer therapy without inducing detrimental systemic side effects. Zinc oxide or TiO_2_ is a semiconducting photosensitizer showing great potential for treating tumor cells in photodynamic therapy (PDT) [38,165]. Under UV irradiation, ROS induced by photogenerated electron–hole pairs on nZnO are effective to treat tumor cells located on or just below the skin [39,165,166,167,168,169,170]. During the therapy, a photosensitizer is first injected into human body, followed by its accumulation in tumor tissues and subsequent UV irradiation for generating ROS (Figure 30) [171]. ZnO NPs induce a preferential killing of cancer T cells with 28–35 times sensitivity relative to normal T cells [161]. So, ZnO NPs can produce more ROS in cancer cells than normal cells due to their faster metabolic rate. In general, photosensitizers should be free from toxic chemical residuals to avoid adverse effects in human tissues. Green ZnO NPs synthesized from the plant extracts containing no harmful surfactant and harsh solvent residuals are particularly useful for PDT treatment.

**Figure 31:**
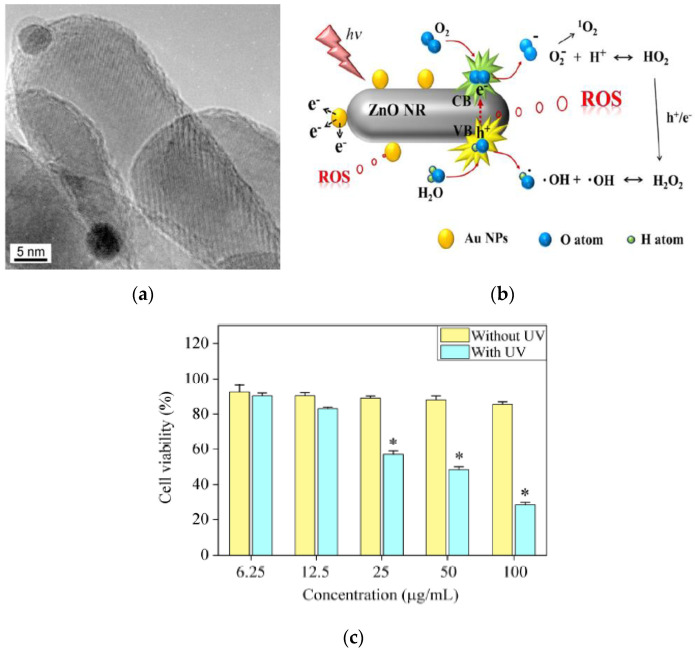
(**a**) TEM image of ZnO:AuNPs nanohybrid. Dark grey particles: AuNPs. (**b**) A schematic showing a high yield production of ROS on ZnO:AuNPs under UV irradiation. (**c**) MTT cell viability of HeLa cells treated with ZnO:AuNPs (molar ratio 20:1) of different concentrations with or without UV irradiation. Data are expressed as mean ± SD, n = 3, * denotes a statistical difference between HeLa cells with and without UV irradiation. Reproduced from [167] with permission of Springer Nature.

Li et al. studied anticancer activity of ZnO NPs (20 nm and 60 nm) with doses of 1.56 and 25 µg/mL under UV irradiation for 180 s on human hepatocarcinoma cells (SMMC-7721) [39]. The cell viability decreased considerably by treating with ZnO NPs of different sizes at a higher dose of 25 µg/mL under UV irradiation. Kang et al. doped ZnO nanorod (NR) with AuNPs in order to suppress electron–hole pair recombination under UV irradiation (Figure 31a) [167]. In comparison to pure ZnO NR, the amount of photoexcited electrons increased markedly on AuNPs-doped ZnO NR, leading to the generation of high ROS yield (Figure 31b). A large amount of ROS production was derived from the generation of photoexcited electrons on the CB of ZnO NR under UV light, with the subsequent flow of those electrons from ZnO to AuNPs prompting the further induction of ROS (Figure 7b). Consequently, ZnO:AuNPs (molar ratio 20:1) hybrid at a dose of 100 µg/mL reduced the viability of HeLa cells to 28% under UV irradiation for 2 min (Figure 31c). It is worth noting that ZnO:AuNPs nanohybrids are capable of producing ROS under visible light. As mentioned previously, AuNPs exhibit localized SPR effect under visible light (Figure 6) [69,70,72]. In this respect, ZnO:AuNPs hybrids show a great potential for cancer treatment in PDT due to the creation of ROS under visible light (Figure 7a).

**Figure 32:**
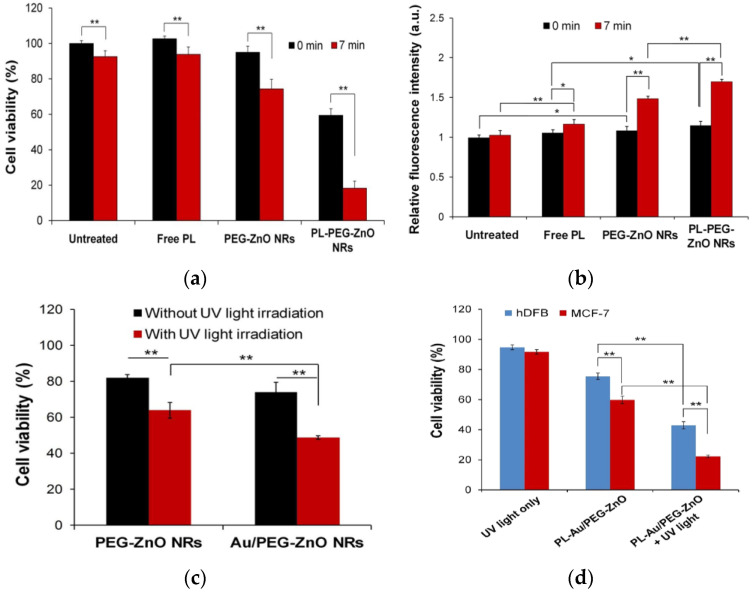
(**a**) MTT assay-cell viability and (**b**) relative ROS generation in MCF-7 cells treated with free PL (0.55 μM), PEG-ZnO NRs (20 μg/mL), and PL-PEG-ZnO NRs (20 μg/mL) with or without UV irradiation for 7 min. * *p* < 0.05 and ** *p* < 0.01. (**c**) Viability of MCF-7 cells treated with PEG-ZnO NRs (20 μg/mL) and Au/PEG-ZnO NRs (20 μg/mL) with or without UV irradiation; ** *p* < 0.01. (**d**) PL-Au/PEG-ZnO NRs induce selective killing in MCF-7, but not in hDFB cells in the absence and presence of UV light (** *p* < 0.01). Reproduced from [169] with permission of the American Chemical Society.

Very recently, Hong et al. studied the anticancer activity of ZnO NRs, polyethylene glycol (PEG)-coated zinc oxide nanorods (PEG-ZnO NRs) and AuNP-coated PEG-ZnO NRs (Au/PEG-ZnO NRs) against MCF-7 human breast cancer cells in the presence and absence of UV light. Some PEG-ZnO NRs were further loaded with antioxidant/anticancer drug, piperlongumine (PL), being designated as PL-PEG-ZnO NRs [169]. Figure 32a shows the viability of MCF-7 cells treated with PL (0.55 µM), PEG-ZnO NRs (20 µg/mL) and PL-PEG-ZnO NRs (20 µg/mL) with or without UV irradiation for 7 min. Comparing to PEG-ZnO NRs, the cell viability declines markedly for PL-PEG-ZnO NRs in the absence of UV light. The increased cytotoxicity is related to the improved cellular uptake of PL by PEG-ZnO NRs. Furthermore, PL-PEG-ZnO NRs also show higher toxicity than PEG-ZnO NRs toward MCF-7 cells under UV irradiation; antioxidant PL contributes to a slightly higher production of ROS (Figure 32b). The modification of PEG-ZnO NRs with AuNPs can further enhance the intracellular ROS level under UV irradiation (Figure 32c). This results from the generation of photoexcited reactive species on ZnO NRs and AuNPs under UV irradiation (Figure 7b). Consequently, PL-Au/PEG-ZnO NRs selectively kill MCF-7 cells, but not normal human dermal fibroblast (hDFB) cells, especially under UV irradiation (Figure 32d).

**Figure 33:**
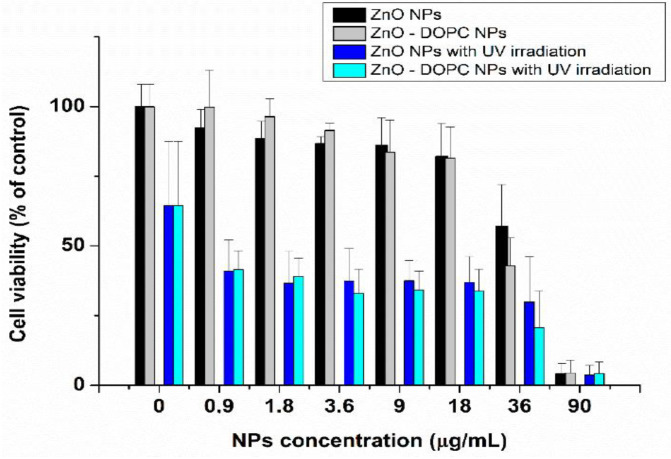
Effect of ZnO NPs and lipid coated ZnO NPs of different concentrations with and without UV irradiation (30 s) on HeLa cell viability. Reproduced from [170] under the Creative Commons Attribution license.

Ancona et al. employed bare ZnO NPs (20 nm) and lipid-coated ZnO NPs for treating HeLa cancer cells under UV irradiation [170]. ZnO NPs were surface functionalized with phospholipid bilayer to improve their dispersion and colloidal stability in phosphate buffered saline (PBS) solution. Lipid-coated ZnO NPs were prepared by a solvent-exchange method using phospholipid DOPC (1,2-dioleoyl-sn-glycero-3-phosphocholine). The cytotoxic effect of bare and lipid coated ZnO NPs in HeLa cancer cells was depicted in Figure 33 [170]. The viability of HeLa cells treated with both types of ZnO NPs decreased markedly under UV irradiation when compared to the control without ZnO NPs-treatment (0 µg/mL NPs). Moreover, bare and coated ZnO NPs exhibited a cytotoxic effect against HeLa cells at concentrations ≥ 0.9 µg/mL under UV light as a result of the ROS generation.

In general, UV light source at high energy with limited penetration depth for PDT would damage human normal tissues. Successful PDT treatment depends on the type of tumors and proper selection of light source. So, near infrared (NIR) light (700–1300 nm) with a deeper penetration depth into human body has attracted considerable attention for therapeutic purposes [172]. However, prolonged exposure to NIR is also harmful to the adjacent normal tissues due to the heating-induced tissue damage. As such, there has been increasing interest in using a low-power laser that emits specific wavelengths of visible light which can penetrate deeply into human tissues for cancer therapy [173]. In this respect, it is advantageous to use metal-doped ZnO nanohybrids capable of generating ROS under visible light illumination for cancer therapy. As mentioned previously, AgNPs and AuNPs exhibit localized surface plasmon resonance with the frequencies covering most of visible and NIR regions [61,62,63,64]. In particular, chemically stable AuNPs are preferred over AgNPs for PDT. Recently, Luengas et al. explored the use of Mn-doped ZnO NPs for treating B-chronic lymphocytic leukemia (B-CLL) in vitro under visible light [174]. They reported that 0.5% Mn-doped ZnO NPs (14.41 nm) exhibit excellent killing effect for B-CLL cells as a result of the ROS generation associated with the introduction of midgap states below the CB of ZnO.

**Figure 34:**
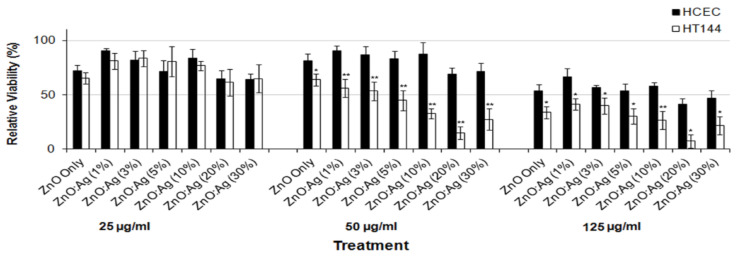
Cell viability of HCEC and HT 144 cells treated with ZnO:Ag nanoparticles of various AgNPs contents and different dose concentrations (25, 50 and 100 µg/mL) for 24 h using SRB assay. Data are presented as mean ± standard deviation. * *p* < 0.001, ** *p* < 0.0001 (two tailed *t*-test) when compared to HCEC. Reproduced from [175] under the terms of the Creative Commons Attribution license.

**Figure 35:**
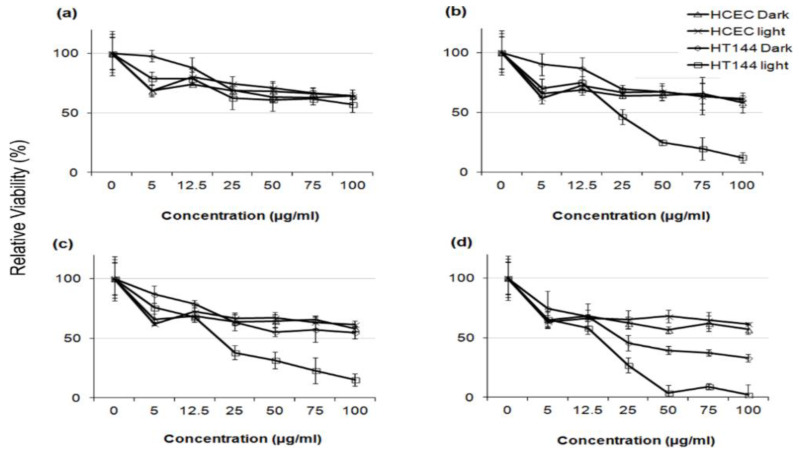
Cell viability of HCEC and HT 144 cells treated with (**a**) ZnO, (**b**) ZnO:Ag(10%), (**c**) ZnO:Ag(20%) and (**d**) ZnO:Ag(30%) of different concentrations (5, 12.5, 25, 50, 75, 100 μg/mL) under visible light and dark conditions. Data are presented as mean ± standard deviation of two independent experiments with triplicates of each sample. Reproduced from [175] under the terms of the Creative Commons Attribution license.

**Figure 36:**
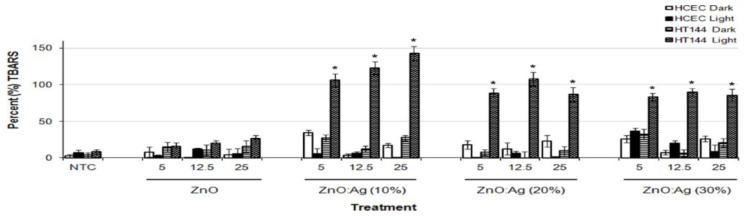
Thiobarbituric acid reactive substances (TBARS) assay results for HCEC and HT 144 cells treated with ZnO, ZnO:Ag(10%), ZnO:Ag(20%) and ZnO:Ag(30%) of different concentrations (5, 12.5 and 25 μg/mL). Data are expressed as percent (%) TBARS (mean ± SD). * *p* < 0.0001 (two tailed *t*-test) when compared to HCEC dark, HCEC light and HT144 dark. Reproduced from [175] under the terms of the Creative Commons Attribution license.

There exists very scarce information in the literature related to the anticancer activity of visible-light-active noble-metal doped ZnO NPs for PDT. Recently, Arooj et al. synthesized ZnO:Ag nanocomposites containing 1%, 3%, 5%, 10%, 20%, and 30% AgNPs using the co-precipitation method. The photocatalytic cytotoxic effects of ZnO:Ag nanohybrids under visible light in human malignant melanoma (HT 144) and human corneal epithelial cells (HCEC) were investigated. ZnO:Ag hybrids destroyed HT 144 cells more effectively than normal HCEC under visible light. The hybrids doped with higher AgNPs contents, i.e., 10%, 20%, and 30% were more toxic than their counterparts with lower AgNPs contents (1%, 3%, and 5%) [175]. Figure 34 shows the viability of HCEC and HT 144 cells treated with ZnO:Ag of various AgNPs contents. Sulforhodamine (SRB) assay is used to assess cytotoxicity due to its simplicity, rapidity and accuracy [176]. At a low dose of 25 µg/mL, there exits little differences between the viability of HCEC and HT 144 cells. However, selective killing of HT 144 cells is observed by treating with ZnO:Ag hybrids at a dose of 50 µg/mL, especially at high AgNPs dopant concentrations ≥ 5%. At this stage, the viability of HT 144 cells falls below 50%. Moreover, AgNPs dopant also releases Ag^+^ ions showing cytotoxicity against both normal and cancer cells. At a high dose of 125 µg/mL, synergistic killing effect of Zn^2+^ and Ag^+^ ions leads to a dramatic reduction in the viability of normal and cancer cells. Figure 35a shows the photocatalytic toxicity of pure ZnO NPs of various concentrations to HCEC and HT 144 cells under visible light and dark conditions. The viability of HT 144 cells decreases with increasing ZnO NPs concentrations. In the dark condition, ZnO NPs can induce a certain amount of ROS due to the presence of intrinsic oxygen vacancies. The oxygen vacancies play a crucial role in the production of ROS for inducing cytotoxicity. In particular, superoxide anion can be produced in an aqueous suspension of ZnO NPs in the dark owing to the interaction of electron from singly ionized oxygen vacancy with adsorbed oxygen molecule [24]. It is noticed that the viability of HT 144 cells reduces remarkably by treating with ZnO:Ag(10%) of various concentrations under visible light (Figure 35b). The AgNPs dopant facilitates the formation of •O^2−^ and •OH radicals on ZnO due to the plasmonic oscillation of free electrons under visible light as mentioned previously (Figure 7a) [69,70,71,72]. So, the ROS production efficacy of ZnO:Ag(10%) is considerably higher than that of pure ZnO under visible light illumination. The enhanced ROS yield of ZnO:Ag (10%) in turn increases its anticancer performance. The anticancer activity of ZnO:Ag(10%) with increasing dose concentrations in the dark condition results from the dissolved Zn^2+^ and Ag^+^ ions as well as oxygen vacancy-mediated ROS generation. Similar visible light-induced phototoxicity due to the ROS generation can be seen in HT 144 cells by treating with ZnO:Ag(20%) and ZnO:Ag(30%) (Figure 35c,d). The ROS can interact with cellular biomolecules such as lipids and DNA. So, oxidative stress is produced due to the deterioration of cell membrane lipids. The extent of lipid peroxidation can be evaluated by measuring MDA level in the cells treated with ZnO:Ag nanoparticles. MDA level is a well-known biomarker of oxidative stress. Figure 36 shows the induction of oxidative stress in HCEC and HT 144 cells by ZnO:Ag nanoparticles through the evaluation of MDA levels using thiobarbituric acid reactive substance (TBARS) assay. Apparently, HT 144 cells treated with ZnO:Ag nanocomposites exhibit very high MDA levels under visible light due to LPO. The elevated MDA levels can damage cells and trigger apoptosis accordingly.

## 5. In Vivo Animal Model

ZnO NPs in the air easily enter human respiratory system through inhalation. The nanoparticles can deposit on alveolar epithelial cells and induce pulmonary inflammatory responses accordingly. The administration routes, such as inhalation, intratracheal instillation, and pharyngeal aspiration, are typically used for evaluating cytotoxicity induced in the lungs of animal models resulting from the uptake of airborne nanoparticles. Conflicting literature results are reported relating in vivo pulmonary exposure of ZnO NPs in the mice. Some workers reported that ZnO NPs cause short-term or transient pulmonary inflammation [177,178], while others indicated that ZnO NPs induce chronic inflammation, eosinophilic/fibrotic/granulomatous inflammation, and bronchocentric pulmonary fibrosis [31,124,179].

**Figure 37:**
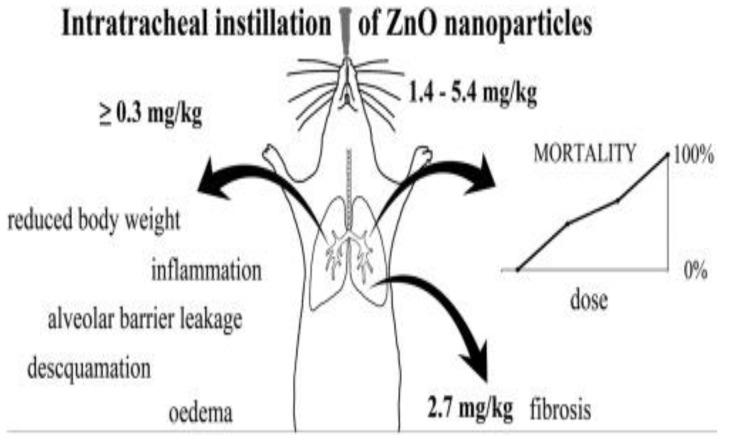
Pulmonary toxicity and mortality in mice administered with nZnO of different sizes, shapes and doses. Intratracheal instillation of ZnO NPs with doses ≥0.3 mg/kg leads to acute pulmonary inflammation (left panel). Pharyngeal aspiration of high ZnO rod doses (≥1.4 mg/kg) leads to a dose dependent mortality in the mice (right panel). Reproduced from [180] under the Creative Commons Attribution license.

Adamcakova-Dodd et al. exposed C57/Bl6 mice to ZnO NPs (10 nm) aerosols for sub-acute inhalation (3.5 mg/m^3^, 4 h/day) for a period of two weeks The inhalation of aerosol nanoparticles led to an increase of macrophages in bronchoalveolar lavage (BAL) fluid, and the induction of cytokines IL-12(p40) and MIP-1α. However, no significant histopathological changes in the lungs were observed. Furthermore, an elevated concentration of Zn was found in the lungs and BAL fluid, demonstrating the dissolution of ZnO NPs in the respiratory system after inhalation [177]. Morimoto et al. also reported a transient increase in total cell and neutrophil numbers in BALF at day 3 of intratracheal instillation [178]. Jacobsen at al. administered ZnO NPs (35 nm) into F344 rats through inhalation and intratracheal instillation [180]. For inhalation experiments, rats were exposed to ZnO NPs (2 and 10 mg/m^3^, 6 h/day) aerosols for four weeks. The rats were dissected at three days, one month, and three months after the experiments; the doses used were 12.5, 25, 50, and 100 μg. The aspiration of high ZnO rod doses (25 up to 100 μg; 1.4–5.4 mg/kg) led to a dose dependent mortality in the mice. However, mice that survived a high dose (50 μg; 2.7 mg/kg) had lung fibrosis due to pulmonary collagen accumulation (Figure 37; right panel). Intratracheal instillation of ZnO NPs of lower doses (≥6 μg; ≥0.3 mg/kg) resulted in acute pulmonary inflammation, leading to reduced body weight, desquamation of epithelial cells of bronchioles, alveolar barrier leakage, and oedema (Figure 37; left panel). By pharyngeal aspiration of ZnO NPs, acute and chronic pulmonary inflammation in rat lungs were reported by Sehsah et al. very recently [181].

Cho et al. administered ZnO NPs (10.7 nm) with doses of 50 or 150 cm^2^/rat into female Wistar rats intratracheally. They reported that ZnO NPs induce diverse pathological changes in the lungs including eosinophilia, airway epithelial cell injury, regenerative goblet cell hyperplasia, and bronchocentric pulmonary fibrosis. Those adverse effects were caused by zinc ions due to the dissolution of ZnO NPs inside lysosomes [124]. Very recently, Wang et al. instilled ZnO NPs (48 nm) into male ICR mice intratracheally at doses of 0.2, 0.4, and 0.8 mg/kg. The control group was treated with phosphate buffer saline (PBS). After instillation, the toxic effects of ZnO NPs on treated mice were monitored for one week [31]. Exposure to ZnO NPs led to bodyweight loss, high levels of MDA and nitric oxide in the lung homogenates, acute and chronic inflammation, and fibrosis. The lung tissue of control group showed a typical spongy appearance with numerous alveoli, and separated with their neighbor cells by thin interalveolar septa (alveolar walls). By treating the mice with 0.2 mg/kg ZnO NPs, thickened interalveolar septa with collapsed alveoli were developed in the lung tissue. Furthermore, thickened interalveolar septa, fibrosis and chronic inflammation were found in the lung tissue of mice upon exposure to ZnO NPs with doses ≥0.4 mg/kg.

**Figure 38:**
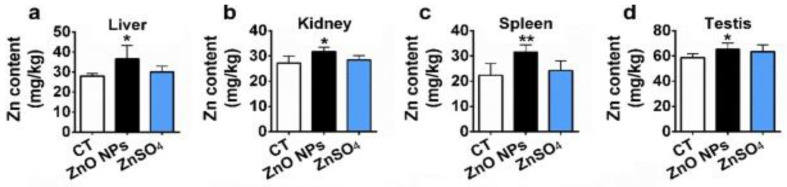
Zn accumulation in target organs of rats exposed intranasally to ZnO NPs with a single dose of 2.56 mg Zn per rat. A same dose of ZnSO_4_ or sodium carboxymethyl cellulose solution (CT, control) was treated to the rats. Data are expressed as mean ± SD (n = 10). * *p* <0.05 and ** *p* < 0.01 imply significant difference compared with CT. Reproduced from [183] under the Creative Commons Attribution license.

**Figure 39:**
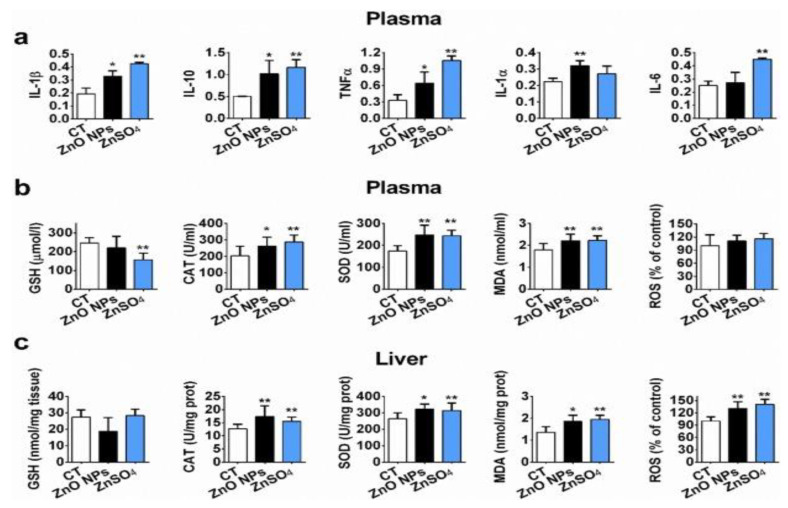
Toxicity of ZnO NPs and ZnSO_4_ to Sprague-Dawley rats via intranasal exposure. (**a**) The induction of inflammatory cytokines in plasma, (**b**) elevated ROS, MDA, CAT and SOD activities in plasma, and (**c**) overexpression of ROS, MDA, CAT and SOD levels in liver. Data are expressed as mean ± SD (n = 10). CT indicates control group without ZnO NPs or ZnSO_4_ treatment. * *p* < 0.05 and ** *p* < 0.01 imply significant difference compared with CT. Reproduced from [183] under the Creative Commons Attribution license.

Kao et al. exposed SD rats to ZnO vapor (38 nm; 2.1 × 10^6^ particles/cm^3^, 6 h/day), and reported that ZnO NPs remarkably increased zinc level in the olfactory bulb. In their study, ZnO NPs were prepared by heating zinc powder in a furnace at 570–600 °C. ZnO vapor was obtained by reacting zinc vapor with oxygen in a high purity nitrogen gas. The vapor was then mixed with filtered air in a series of cooling and humidifying steps [182]. Following nasal exposure, ZnO NPs entered olfactory bulb in the brain. This implied that airborne ZnO NPs would enter the central nervous system (CNS) via an olfactory bulb–brain translocation pathway, resulting in neurotoxicity. Very recently, Guo et al. intranasally administered spherical ZnO NPs (31 ± 5 nm) or ZnSO_4_ to male Sprague Dawley rats with a single dose of 0.85, 1.7, or 2.56 mg per rat. The rats were left undisturbed for seven days post exposure, and thereafter sacrificed accordingly [183]. Intranasal exposure of ZnO NPs of 2.56 mg per rat led to an accumulation of Zn mainly in the lung, liver, kidney, spleen, and testis. Zinc sulfate was used to assess the toxic effects of released Zn^2+^ ions after intranasal exposure. No accumulation of Zn was detected in liver due to ZnSO_4_ treatment (Figure 38). Both ZnO NPs and ZnSO_4_ exposures induced proinflammatory responses including interleukin 1 alpha (IL-1α), interleukin 1 beta (IL-1β), IL-10, IL-6, and tumor necrosis factor alpha (TNFα) (Figure 39a), and caused elevated levels of ROS, MDA, catalase (CAT), and superoxide dismutase (SOD) in blood plasma. However, GSH level in plasma was substantially decreased by ZnSO_4_ treatment (Figure 39b). Overexpression of ROS, MDA, CAT and SOD activities with a reduced level of antioxidant GSH were detected in liver of mice treated with ZnO NPs (Figure 39c).

Sharma et al. orally administered ZnO NPs (30 nm) to Swiss albino mice at doses of 50 and 300 mg/kg body weight (bw) for 14 consecutive days. The nanoparticles were suspended in milli-Q water prior to oral gavage [184]. The oral exposure of ZnO NPs of 300 mg/kg for 14 consecutive days led to a significant accumulation of ZnO NPs in liver as measured by the atomic absorption spectrometry. Moreover, elevated alanine aminotransferase (ALT; an indicator for liver disease) and alkaline phosphatase (ALP) levels in serum were also detected. In contrast, there was no accumulation of Zn in the kidney and brain of ZnO NPs-treated mice when compared to the control. The accumulation of Zn in the liver led to the ROS generation and DNA damage as shown by the lipid peroxidation and Fpg-modified comet assay results. The comet assay indicated a remarkable increase in the Fpg-specific DNA lesions in liver, revealing oxidative stress induced DNA damage and thus causing apoptosis in liver cells. The toxicity was not detected in mice treated at a low dose of 50 mg/kg. In a recent study, Srivastav et al. orally administered ZnO NPs (13–68 nm) suspensions at doses of 300 and 2000 mg/kg bw to Wistar rats for several time points up to 14 days. The liver was found to accumulate more zinc than kidney at time points of 24 h and 48 h. The elevated levels of ALT, ALP, and LDH at a dose of 2000 mg/kg bw for 24 h, 48 h, and 14 days were observed [185]. So, the damage of the liver by ZnO NPs led to a high ALT level. In addition, erythrocytes of mice showed hemolytic activity after oral uptake of 2000 mg/kg ZnO NPs for 48 h.

Yang et al. administered ZnO NPs (80 nm) at doses of 200 mg/kg/day and 400 mg/kg/day to male C57BL/6 mice through oral gavage for 90 consecutive days. They reported that ZnO NPs cause the disruption of ER structure and the function of hepatocytes, resulting in ER stress. As such, ER stress and oxidative stress were responsible for ZnO NPs-induced hepatotoxicity [35]. TEM examination of hepatocytes revealed ER swelling and ribosome degranulation. The levels of mRNA expression of pro-apoptotic genes (CHOP and bax) in the mouse livers treated with ZnO NPs were remarkably higher than those of control mice. Moreover, a dramatic decrease in anti-apoptotic gene bcl-2 level in the livers, especially for the mice treated with a high dose of 400 mg/kg. Caspase proteins, such as caspase 3, caspase 9, and caspase 12, of ZnO NPs treated mice were activated, and much higher than those of control mice. The mRNA expression levels of ER stress-associated genes including grp78, grp94, pdi-3, and xbp-1 were also increased in the livers of mice treated with 200 and 400 mg/kg ZnO NPs [35]. Very recently, Yousef et al. orally administered 100 mg/kg bw ZnO NPs (100 nm) to Wistar rats for 75 consecutive days [186]. Pronounced hepatorenal toxicity and systemic inflammation were reported in ZnO NPs treated mice. The liver and kidney tissues exhibited significantly high levels of p53, TNF-α and IL-6 as compared to the control group. An elevated p53 level implied the induction of apoptosis in liver and kidney tissues of rats treated with ZnO NPs. Those tissues also exhibited very low GSH, but a high level of lipid peroxidation end products (TBARS), demonstrating the induction of oxidative stress.

**Figure 40:**
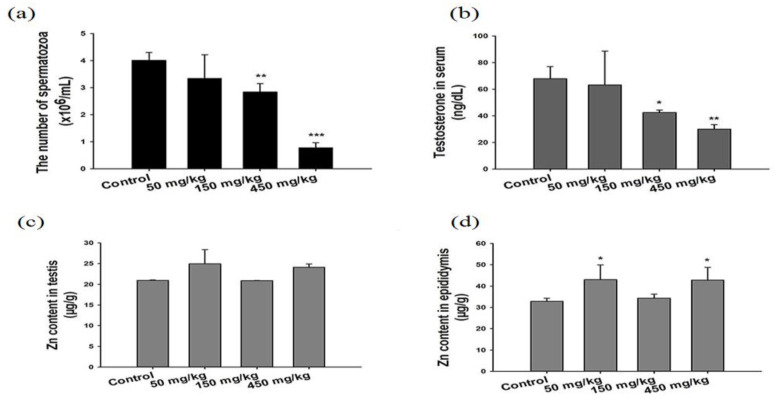
(**a**) Sperm count in the left epididymis, (**b**) testosterone level in serum, (**c**) Zn level in testis and (**d**) Zn level in epididymis of Kunming mice subjected to a 14-day oral administration of the ZnO NPs. Data are expressed as mean ± SD, * *p* < 0.05, ** *p* < 0.01, *** *p* < 0.001 compared with control. Reproduced from [188] under the terms of Creative Commons Attribution license.

**Figure 41:**
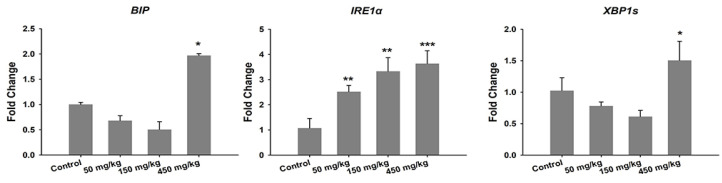

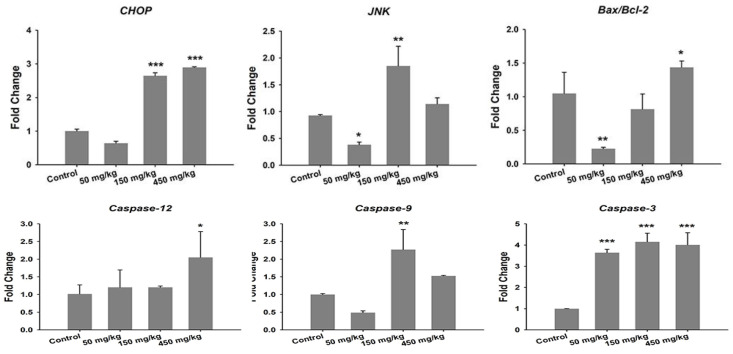
The effects of orally administered ZnO NPs of different doses on mRNA expression levels of ER stress, and apoptosis-related genes as determined by real-time polymerase chain reaction (RT-PCR). The JNK and caspase-12 expression levels in the testis are determined with immunofluorescence assay. The data are presented as mean ± standard deviation. * *p* < 0.05 vs. control, ** *p* < 0.01 vs. control, and *** *p* < 0.001 vs. control. Reproduced from [188] under the terms of Creative Commons Attribution license.

**Figure 42:**
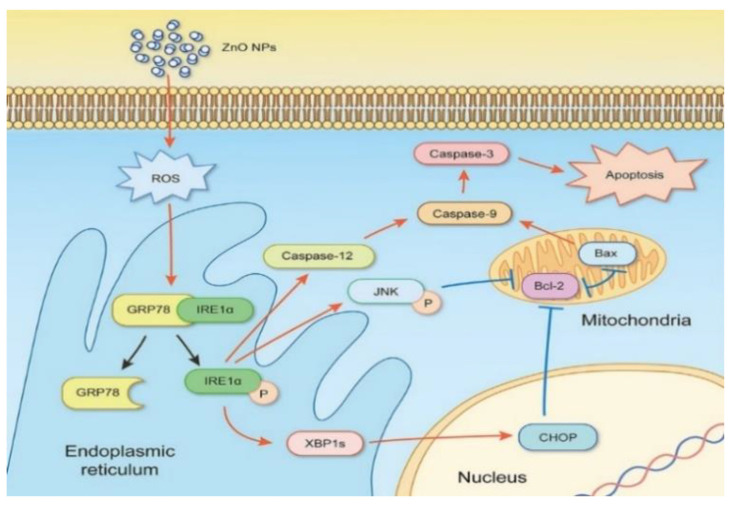
Schematic illustration for activating ER-stress pathway in the testis of male mice treated with ZnO NPs. Simultaneous mitochondrial apoptosis leads to Bax/Bcl2 signaling, caspase 9 and caspase 3 activation. “↑” activation, “⊥” inactivation, and “P” phosphorylation. Reproduced from [188] under the terms of Creative Commons Attribution license.

Apart from hepatotoxicity, ZnO NPs can penetrate through the blood–testis barrier of male mice due to their nanoscale dimension, leading to a reduction in sperm cell population, and the induction of sperm deformity [187]. Very recently, Tang et al. evaluated the toxic effects of ZnO NPs (30 nm) on the reproductive system of male Kunming mice through oral gavage of ZnO NPs with doses of 50, 150 and 450 mg/kg for consecutive 14 days. The mice were sacrificed at 24 h after the last gavage [188]. Figure 40a,b show the number of sperm cell and testosterone hormone level decrease with increasing ZnO NPs doses. The Zn levels in the testis and epididymis are higher in the mice treated with 50 and 450 mg/kg ZnO NPs (Figure 40c,d). From the real time polymerase chain reaction (RT-PCR) results, the gene expressions related to ER stress including unfolded protein responsive protein immunoglobulin-binding protein (BIP/GRP 78), inositol-requiring protein 1α (IRE1α) and X-box-binding protein 1 splicing (XBP1s) levels are upregulated for the mice treated with 150 mg/kg (Figure 41). Furthermore, CHOP, JNK, and bax/Bcl2 protein levels increase significantly at doses ≥150 mg/kg. The CHOP gene is activated by ER stress as mentioned previously, while the Bcl2 protein family is the key regulator of apoptosis of mitochondria. ER stress regulates caspase activation as the functions of ER are severely impaired due to the ROS generation. As a result, caspase 12, caspase 9, and caspase 3 involved in the apoptotic signaling pathway are upregulated (Figure 41). Altogether, ER stress and mitochondrial dysfunction due to the ROS generation lead to final cell apoptosis as schematically illustrated in Figure 42.

**Figure 43:**
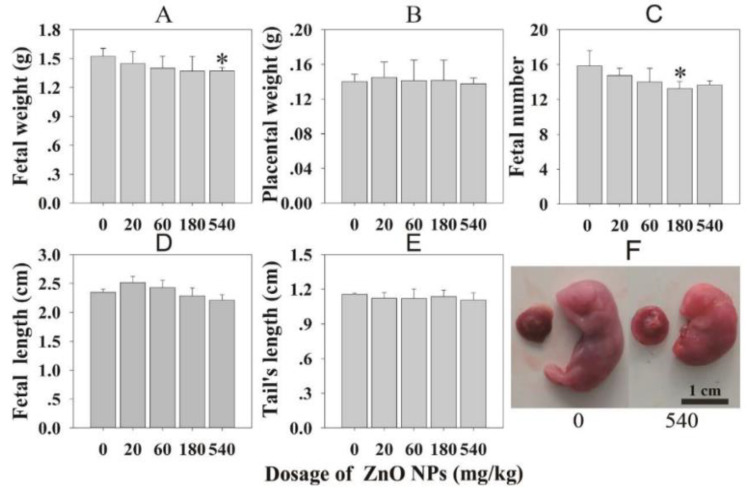
Fetal development in pregnant mice upon oral exposure to ZnO NPs. (**A**) Fetal weight, (**B**) placental weight, (**C**) fetal number, (**D**) fetal length, (**E**) tail length, and (**F**) fetal image showing fetus body malformation in pregnant mice treated with 540 mg/kg ZnO NPs. The data are expressed as mean ± SD (n = 60); * *p* < 0.05 vs. control. Reproduced from [189] under the Creative Commons Attribution license.

It is noted that oral administration of ZnO NPs to female mice also affects their reproductive system. Chen et al. have studied the adverse effects of orally administered ZnO NPs (30 nm) on the fetal development in pregnant Kunming mice. The pregnant mice were treated with ZnO NPs at doses of 20, 60, 180, and 540 mg/kg from gestation day 10.5 to 17.5. The exposure of pregnant mice to spherical ZnO NPs leads to dam injury, fetal growth restriction, fetus number reduction and fetus body malformation (Figure 43) [189]. Moreover, zinc concentration is significantly elevated in the uterus, placenta, and fetus of mice treated with 540 mg/kg ZnO NPs. The RT-PCR and immunocytochemistry results reveal that ZnO NPs induce placenta oxide stress and ER stress. The upregulation of chop and JNK expressions induce ER stress response, and this in turn activates ER-associated caspase 12, leading to placental apoptosis. In another study, they show that N-Acetyl-cysteine (NAC) is an effective antioxidant for reducing reproductive toxicity caused by ZnO NPs. NAC functions as a cellular oxidative stress inhibitor, thus reducing the expression of ATF4, JNK, and Caspase 12 in the placenta. ATF4 activates genes in response to endoplasmic reticulum stress [190].

Ryu et al. conducted a repeated dermal exposure of citrate coated ZnO NPs (20 nm; 250, 500 and 1000 mg/kg bw) to Sprague Dawley rats for 90 days. Transient, dose-dependent irritation of the clipped skin at the application sites was observed [191]. However, no obvious adverse effects of ZnO NPs up to 1000 mg/kg bw applied dermally to the rats. On the contrary, Surekha et al. reported a significant decline in the collagen content of the skins of Sprague Dawley rats treated with ZnO NPs (20 nm) of three different doses (75, 180, and 360 mg/kg bw). ZnO NPs paste was applied to the clipped skin on a 5 day per week basis for 28 consecutive days. The collagen reduction implied that ZnO NPs penetrate deeply into the dermis and damage the collagen layer accordingly [192].

The intraperitoneal (i.p.) administration of ZnO NPs (50 nm; 2.5 g/kg bw) and ZnO microparticles (MPs) into male ICR mice led to their biodistribution and accumulation in liver, spleen, lung, kidney and heart. ZnO NPs produced a remarkable higher Zn concentration in the liver, spleen, and lung compared to ZnO MPs [193]. Hong et al. reported that the i.p instillation of spherical ZnO NPs (20 nm) at a dose of 100 mg/kg bw to male ICR mice resulted in the increase of ALT and ALP levels in serum, demonstrating liver dysfunction or damage [194]. Recently, Abbasalipourkabir et al. studied the toxicity of ZnO NPs (10–30 nm) with dose levels of 50, 100, 150 and 200 mg/kg bw on adult male Wistar rats through intraperitoneal injection [195]. The MDA level in serum increased with increasing ZnO NPs doses, indicating the generation of oxidative stress. High ALT level was detected in the serum at ZnO NPs doses ≥ 50 mg/kg. Furthermore, ZnO NPs reduced sperm number and motility, and increased abnormality in male rats.

Intravenous administration of nanoparticles to mice could lead to their biodistribution and accumulation in several target organs such as liver, kidney and lung [196]. Recently, Fujihara et al. administered intravenously ZnO NPs with doses of 0.05 and 0.2 mg/kg into the tail veins of female ICR mice [197]. Following injection, ZnO NPs circulate in the blood, and reach the target organs. They accumulate mainly in the lung and spleen within 60 min. Furthermore, a dose-dependent increase in 8-hydroxy-2′-deoxyguanosine (8-OHdG) level is observed, demonstrating oxidative damage of DNA in ZnO NPs-treated mice. Han et al. injected ZnO NPs with doses of 1 and 5 mg/kg into male CD-1 mice intravenously. The injection leads to the translocation of ZnO NPs into reproductive organs, thus affecting the functions of testes and epididymis greatly. As such, abnormal sperm morphologies, e.g., double head, small head, and double tail are produced in the treated mice [143]. Choi et al. introduced ZnO NPs (14.4 nm) at doses of 3 and 30 mg/kg bw into Sprague Dawley rats via intravenous injection and oral administration routes [198]. After intravenous injection, Zn peak concentration occurs at 5 min. At day 1, ZnO NPs accumulate in the lung, liver, and kidney of the mice treated with a dose of 30 mg/kg, leading to high Zn contents in those organs, i.e., lung (391.23 µg/g), liver (88.22 µg/g), and kidney (34.63 µg/g). So, the lung is the main target organ of ZnO NPs after injection. At day 7, Zn level in liver reduces to 32.3 µg/g, being close to that of untreated mice (control) of 33.07 µg/g. Similarly, Zn level in kidney reduces to 20.14 µg/g, and slightly lower than that of control mice, i.e., 20.98 µg/g. The reduction of Zn levels in kidney and liver of treated mice is due to the excretion of Zn through urine and feces. The level of Zn excretion in urine increases rises sharply from day 2 to day 7. However, Zn content in the lung remains at a relatively high value at day 7 (i.e., 23.87 µg/g) when compared to the Zn level of control mice of 15.72 µg/g.

On the basis of the results of in vivo mouse model, it appears that ZnO NPs are highly toxic to mammalian cells. The toxicity depends on the size, dose, time, and administration routes. The uptake of ZnO NPs into mice via i.p. instillation and intravenous injection poses a high degree of toxicity due to the accumulation of ZnO NPs in the target organs such as liver, spleen and lung. Moreover, high ALT and low GSH levels in orally administered mice with ZnO NPs reveal the induction of oxidative stress. So, care has to be taken with the increase in the probability of human exposure to nZnO through injection and oral routes. Table 2 summarizes the literature survey results of in vivo studies on a mouse model showing toxic effects of ZnO NPs through different administration routes.

## 6. Conclusions and Outlook

The FDA approves ZnO as a GRAS material having extensive applications in cosmetics, food additives, and healthcare products. However, this approval is mainly designated for ZnO microparticles. With the increasing use of nZnO in cosmetic, biomedical, and industrial sectors, public awareness and concern have been raised about the safety of these nanomaterials. In vitro cell cultivation experiments demonstrate that nZnO exhibit adverse impacts and toxic effects to a number of mammalian cell lines, including human erythrocyte, human dermal fibroblast, human lymphocyte, human keratinocyte, murine fibroblast, murine Leydig cell, murine macrophage, murine retinal ganglion cell, murine stem cell, etc.

Nano-sized ZnO can penetrate easily through mammalian cell walls into cytoplasm, creating ROS that impair ER and mitochondrial functions, interacting with biomolecules such as DNA, lipids, and proteins. These adverse interactions lead to apoptosis by activating Bax, Bcl2, and p53 expressions as well as caspase proteins. nZnO can also enter the cells through endocytosis process. By trafficking into acidic lysosomes, they release intracellular Zn^2+^ ions that degrade the functions of cellular components through the ROS generation. In certain cases, ZnO NPs can dissolve into Zn^2+^ ions extracellularly. Those ions then diffuse into the cytoplasm and induce the generation of ROS and oxidative damage. In addition, ZnO NPs exhibit selective killing ability to cancer cells. ZnO NPs show 28–35 times selective toxicity towards cancer cells relative to normal cells.

ZnO is a semiconductor with a large bandgap showing photocatalytic effect. By irradiating with UV light, photoinduced electron–hole pairs create ROS by reacting with adsorbed oxygen or water molecules. The photogenerated ROS selectively induce apoptosis in cancer cells, thus showing potential application in photodynamic therapy. However, the therapeutic application of ZnO NPs in PDT is limited by their inability to generate ROS under visible light. Doping ZnO NPs with noble metals and transition metals increases their spectral response in the visible region, and facilitates the formation of ROS under visible light. In particular, noble metal dopants such as AuNPs and AgNPs exhibit plasmonic oscillation under visible light illumination. The hot electrons from noble metal NPs move to the conduction band of ZnO, thereby generating superoxide radical through a reaction with adsorbed oxygen molecule. The ZnO:Ag nanocomposites have been reported to exhibit selective killing against human malignant melanoma (HT 144) than normal human corneal epithelial cells (HCEC) [175]. However, AgNPs also exhibit adverse effect to human cells through the release of Ag^+^ ions [8]. Therefore, chemically stable AuNPs are more suitable to dope ZnO NPs for generating ROS under visible light for PDT. As mentioned, ZnO NPs prepared from hydrothermal/solvothermal synthesis and sol–gel often contain stabilizing agents or surfactants for precise control over their sizes and shapes. Those surfactants are toxic and harmful to human tissues. So, biosynthesized ZnO NPs free from surfactants can be used as a photosensitizer for PDT, and injected into the human body for killing cancer cells. More studies are needed to assess the use of green ZnO:Au hybrids as the next-generation photosensitizer under visible light for cancer therapeutic applications.

Finally, in vivo studies on a mouse model showing the toxic effects of ZnO NPs on internal organs through different administration routes. The toxicity depends on the size, dose, time, and administration routes. In particular, the administration of ZnO NPs into mice via i.p. instillation and intravenous injection poses a high degree of cytotoxicity due to the accumulation of ZnO NPs in the target organs, such as the liver, spleen, and lung. Accordingly, the use of ZnO NPs should be carefully considered, especially for PDT that requires the injection of ZnO NPs-based photosensitizer into human body.

## Figures and Tables

**Table 1:** Toxic effects of ZnO nanostructures to normal mammalian cells and cancer cells.

Shape & Size	Cell Type	ZnO Dose & Exposure Time	Cytotoxic Effect	Ref.
***Normal cells***				
Polygonal nanoparticles (NPs); 55 nm	Human HaCaT cells & gingival fibroblasts	10, 15, 30 and 100 µg/mL for 24 h	No toxicity up to 15 µg/mL ZnO NPs. Viability < 50% at a dose of 100 µg/mL	[135]
Tetrapods (length: 500 nm–50µm; thickness: 200 nm–2µm); Spherical NPs (150 nm)	Human dermal fibroblasts	15.2, 25.3 and 35.5 µg/mL for 24 h	Tetrapods are relatively less toxic than ZnO NPs that release more Zn^2+^ ions. The minimal size of tetrapods and diameter of NPs fall in sub-micrometer scale.	[53]
Spherical NPs; 22 nm	Human HaCaT cells	10, 20, 40 and 80 μg/mL for 24 h.	Dose-dependent toxicity. Mitochondrial dysfunction, lactase dehydrogenase (LDH) leakage, and reactive oxygen species (ROS) generation	[136]
Nanorods; 47.8–52.5 nm	Human erythrocytes	50, 100, 250 and 500 ppm for 1 h at 37 °C	Lipid peroxidation of cell membrane, causing hemolysis; ROS generation	[140]
Polygonal NPs; 60 nm	Murine retinal ganglion cells	0.63, 1.25, 2.5, 5, & 10 µg/mL for 24, 48, 72 h	Dose-dependent toxicity. Excessive ROS creation leads to overexpression of caspase-12 and final cell death	[123]
Commercial NPs	Murine GC-1 spg cells	1, 2 and 4 µg/mL	Dose-dependent toxicity. Induced autophagy due to elevated microtubule-associated proteins 1A/1B light chain 3-II (LC3-II) & Beclin1 levels. Elevated levels of BcL2-associated X protein (Bax), caspase 3 and caspase 8	[49]
Spherical NPs; 20–110 nm	Murine Leydig & Sertoli cells	5, 10, 15 and 20 µg/mL for 12 h and 24 h	Internalization of ZnO NPs. Dose- and time-dependent toxicity. Apoptosis at doses ≥ 15 µg/mL due to LDH & ROS	[143]
Nanorods (width: 15.38 nm; length: 82.34 nm)	Murine JB6 Cl 41-5a skin cells	5, 10 and 20 µg/mL for 24, 48 and 72 h	Mitochondria dysfunction, dose-and time-dependent ROS generation	[137]
Nanorods (width: 40 & 80 nm; length: 80–250 nm); NPs (20 nm)	Murine L929 fibroblasts	0.1, 0.2, 1, 3, 5 & 7 mM for 24, 48 and 72 h	Size- and dose-dependent toxicity. Shape changes from elongated to rounded forms at a ZnO dose of 5 mM	[144]
Nanorods; 45 nm	Primary rat astrocytes	4, 8 and 12 µg/mL for 6, 12 and 24 h	Reduce cell viability, increase LDH and ROS levels, activate caspase 3 in a dose- and time-dependent manner	[139]
***Cancer cells***				
Polygonal NPs; 30 nm	HepG2	0.8, 2, 8, 14 and 20 µg/mL for 6 h, 12 h & 24 h	Dose- and time dependent toxicity. Cell viability decreases while LDH rises at doses ≥14 mg/mL for 12 h & 24 h Induce ROS, so reducing mitochondrial membrane potential (MMP), and activating Bax, Bcl2, p53 & caspase 9	[50]
Polygonal NPs; 21.34 nm	HepG2, A549 & primary rat cells	5, 10 & 15 µg/mL for 24 h	Selective killing to HepG2, A549 by inducing dose-dependent cytotoxicity. Upregulation of Bax and p53 gene levels, and downregulation of Bcl2	[148]
Spherical NPs; 13 nm	MCF-7 & HepG2	2.5, 5, 10, 25, 50 & 100 µg/mL for 24 and 48 h	Dose-and time-dependent toxicity. Apoptosis due to ROS production, upregulation of Bax, p53 & caspase 3	[150]
Polygonal; 10–59 nm	MCF-7	62.5, 125, 250, 500, and 1000 µg/mL for 24 h	ZnO NPs arrested the cell cycle in the G2/M phase. Upregulation of p53, p21, & Bax, and downregulation of Bcl2, and extracellular regulated kinases (ERK1/2) in a dose-dependent manner	[152]
Spherical NPs; 15–18 nm	A549	0.1, 10 and 100 µg/mL for 4, 24 and 48 h	Uptake of extracellular Zn^2+^ ions induce ROS and deoxyribonucleic acid (DNA) damage	[122]
Spherical NPs; 30, 80 & 200 nm	THP-1 monocytes & macrophages	1, 10, 25, 50, 75, and 100 µg/mL	Size- and dose-dependent toxicity. Intracellular dissolution of NPs yield Zn^2+^ ions & mitochondrial superoxide	[126]
Spherical NPs; 25–40 nm	HEK293	5, 15, 25, 50, 75 and 100 µg/mL for 3, 24 & 48 h	Dose-and time-dependent toxicity. Generation of ROS and oxidative stress, causing MMP reduction, loss of lysosomal activity, and apoptosis	[147]
Polygonal NPs; 20 nm	SKOV3	5, 10, 20 and 30 μg/mL for 12 h & 24 h	Dose- and time-dependent toxicity. Apoptosis at doses ≥20 μg/mL through the ROS creation, MMP reduction, upregulation of p53, Bax, caspase 9, & downregulation of Bcl2	[156]
Polygonal NPs; 55 nm	Ca9-22	10, 15, 30 and 100 μg/mL for 24 h	Apoptosis at doses ≥30μg/mL due to ROS creation, MMP reduction and DNA fragmentation	[135]
Hexagonal prismatic NPs; 18.5 and 47.1 nm	SHSY5Y	5, 10, 20, 40, 60, 80 & 100 µg/mL for 2, 6, 12 and 24 h	Size- and time-dependent toxicity. Intracellular Zn^2+^ ions induced the generation of ROS and oxidative stress, leading to mitochondria dysfunction, cytoskeletal disruption, and apoptosis	[154]
Spherical QDs; 7.10 nm	HEK 293T & HeLa	25, 50,100, 200 & 400 µg/mL for 6, 24, 48 and 72 h	Dose- and time-dependent cytotoxicity. ROS production in both cell types in a time- and dose-dependent manner, causing MMP reduction	[162]
Nanorods (width: 40 & 80 nm; length: 80–250 nm); NPs (20 nm)	HeLa	0.05, 0.1, 0.2, 1, 2, 3,4 & 5 mM for 24, 48 and 72 h	Size- and dose-dependent toxicity. ROS generation and released Zn^2+^ ions are responsible for apoptosis	[144]

**Table 2:** ZnO NPs induced toxicity in a mouse model through different administration routes.

Administration Route	ZnO NPs Dose & Test Time	Cytotoxic Effect	Ref.
Aerosol inhalation	3.5 mg/m^3^ at 4 h/day for 2 wks	An increase of macrophages in bronchoalveolar lavage (BAL) fluid	[177]
Aerosol inhalation	2 and 10 mg/m^3^, 6 h/day for 4 wks. The rats were sacrificed at 3 d, 1 mo & 3 mos after the inhalation exposure	An increase of macrophages, neutrophils, cytokines and LDH levels in BAL fluid after 3d post-inhalation exposure. Those values return to normal compared to control after 1–3 months post-inhalation exposure	[178]
Nasal exposure	2.1 × 10^6^ particles/cm^3^ at 4 h/d for consecutive 3 d	ZnO NPs enter olfactory bulb and induce neurotoxicity	[182]
Intranasal exposure	A single dose of 0.85, 1.7 and 2.56 mg/rat. Rats were sacrificed at 7 d post-exposure.	Biodistributed in liver, kidney, spleen and testis. Induction of inflammatory cytokine responses like interleukin 1 alpha (IL-1α), IL-1β, IL-10, IL-6, and tumor necrosis factor alpha (TNFα). Upregulation of ROS & malondialdehyde (MDA) in blood plasma and liver, and depletion of GSH level in liver	[183]
Intratracheal instillation	0.8 and 4 mg/kg. Monitoring toxic effects after instillation for 3 d, 1 wk, 1 mo, 3 mos, and 6 mos	Transient increase in total cell and neutrophil numbers in BALF at day 3	[178]
Intratracheal instillation	≥0.3 mg/kg. Monitoring toxic effects after instillation for 1 d, 3 d and 7 d	Acute pulmonary inflammation, reduced body weight, desquamation, alveolar barrier leakage and oedema	[180]
Intratracheal instillation	50 and 150 cm^2^/rat. Monitoring toxic effects after instillation for 24 h, 1 wk, and 4 wks	Eosinophilic/fibrotic/granulomatous inflammation, and bronchocentric pulmonary fibrosis	[124]
Intratracheal instillation	0.2, 0.4 and 0.8 mg/kg. After instillation, monitoring toxic effects for 7 d	Reduced body weight, increased MDA level in lung homogenates. Chronic inflammation and pulmonary fibrosis at doses ≥ 0.4 mg/kg ZnO NPs	[31]
Oral gavage	200 and 400 mg/kg/day for consecutive 90 d	High alanine transaminase (ALT) and low glutathione (GSH) levels. Endoplasmic reticulum (ER) swelling in hepatocytes as revealed by transmission electron microscopy. Oxidative stress and ER stress involve in liver injury, triggering caspase 3, caspase 9 and caspase 12, and up-regulation of C/EBP homologous protein (CHOP) and bax expressions	[35]
Oral gavage	50 and 300 mg/kg for consecutive 14 d	Elevated ALT and ALP levels in serum, and accumulation of ZnO NPs in liver at a high dose of 300 mg/kg, causing ROS production and DNA damage in liver. No toxicity at a low dose of 50 mg/kg	[184]
Oral gavage	300 and 2000 mg/kg for 24 h, 48 h and 14 d	High ALT, ALP and LDH levels at a dose of 2000 mg/kg at all time points; hemolytic activity for this dose at 48 h	[185]
Oral administration	100 mg/kg for consecutive 75 d	Hepatorenal toxicity due to high liver and kidney levels of p53, TNF-α and IL-6. Generation of oxidation stress as shown by low GSH but high thiobarbituric acid reactive substance (TBARS) levels	[186]
Oral gavage	50, 150 and 450 mg/kg for 14 d	Reduction of sperm cell count and testosterone in testes. Upregulation of ER stress gene proteins. Activation of caspase proteins	[188]
Oral administration	20, 60, 180, and 540 mg/kg to pregnant mice from gestation day 10.5 to 17.5	High Zn level in uterus, placenta & fetus. ZnO NPs induce dam injury, restrict fetal growth, and reduce fetus number. Upregulation of CHOP & c-Jun N-terminal kinase (JNK) levels in placenta, activating ER-associated caspase-12 & inducing apoptosis	[189]
Dermal exposure	250, 500 and 1000 mg/kg treated dermally for 90 d	No obvious adverse effects of ZnO NPs up to 1000 mg/kg bw	[191]
Dermal exposure	75, 180 and 360 mg/kg for 28 d	ZnO NPs penetrate into the dermis and reduce its collagen content	[192]
Intraperitoneal injection	Single dose of 2500 mg/kg. Monitoring toxic effects after injection for 24, 48 and 72 h	Accumulation in liver, spleen, lung, kidney and heart	[193]
Intraperitoneal injection	1, 10 and 100 mg/kg. Monitoring toxic effects after injection for 1 d, 7 d and 14 d	High ALP and ALT levels in the serum upon injection of ZnO NPs with a dose of 100 mg/kg	[194]
Intraperitoneal injection	50, 100, 150 and 200 mg/kg. Monitoring toxic effects after injection for 10 d	High MDA and ALT levels in serum. ZnO NPs reduce sperm number and motility	[195]
Intravenous injection	1 mg/kg and 5 mg/kg. Monitoring toxic effects after injection for 7 d and 21 d	Abnormal sperm morphologies (double head, small head, double tail)	[143]
Intravenous injection	0.05 and 0.2 mg/kg. Monitoring toxic effects after injection for 1 d, 3 d and 6 d	Accumulation of ZnO NPs in lungs and spleen. Oxidative damage of DNA	[197]
Intravenous injection	3 mg/kg and 30 mg/kg. Monitoring toxic effects after injection for 1 d, 2 d and 7 d	Accumulation of ZnO NPs in lung, liver and kidney at day 1. Zn level remains high in lung at day 7	[198]
